# miRNA/mRNA analysis of increased TGF-β pathways drive epithelial-mesenchymal transition and regulatory T cell differentiation

**DOI:** 10.1101/2025.06.23.661210

**Published:** 2025-06-26

**Authors:** Toni Darville, Xuejun Sun, Yu Zhang, Catherine M. O’Connell, Neha Mokashi, Weiming Tang, Aakash Bhardwaj, Bryce Duncan, Charles W. Andrews, Harold Weisenfeld, Xiaojing Zheng

**Affiliations:** 1. Department of Pediatrics, University of North Carolina at Chapel Hill, Chapel Hill, NC; 2. Department of Biostatistics, University of North Carolina at Chapel Hill, Chapel Hill, NC; 3. Department of Medicine, University of North Carolina at Chapel Hill, Chapel Hill, NC; 4. Pathology consultant, Newburyport, MA; 5. The University of Pittsburgh School of Medicine and the Magee-Womens Research Institute, Pittsburgh, PA

## Abstract

*Chlamydia trachomatis* genital tract infection is linked to severe reproductive complications in women, including ectopic pregnancy, infertility, and adverse pregnancy outcomes. Mouse models of infection suggest that chlamydia-induced dysregulation of microRNAs (miRNAs) can drive harmful cytokine responses, pathogenic epithelial-mesenchymal transition (EMT), and fibrosis. To investigate these mechanisms in humans, we profiled miRNA and mRNA expression in endometrial biopsies from women with endometrial infection (Endo+) and compared them to profiles from women with cervix-only infection (Endo−) or no infection. Ingenuity Pathway Analysis (IPA) revealed that Endo+ tissues had upregulated genes associated with innate and adaptive immune response pathways, as well as EMT regulation, while downregulated genes were linked to cell cycle control. An integrative miRNA-mRNA analysis, which combined a review of published miRNA regulation in human infections and immune responses with IPA’s miRNA target filter, identified differentially expressed miRNAs that modulate these pathways in the endometrium of Endo+ women. Functional annotation of these miRNAs showed a predominance of downregulated miRNAs that typically suppress EMT and regulatory T cell (Treg) differentiation, along with miRNAs that usually enhance Th17 responses. Comparisons with previously identified mRNA pathways in blood samples from women with endometrial *Chlamydia* infection indicated that alterations in TGF-β signaling and EMT were specific to the endometrium. Overall, the miRNA-mRNA interactions inferred from Endo+ tissue suggest increased activity in TGF-β pathways that promote enhanced EMT and Treg differentiation, while reducing Th17 activation. These changes highlight a dual potential for promoting tissue scarring while dampening inflammatory responses that could otherwise limit infection.

## Introduction

Sexually transmitted *Chlamydia trachomatis* (Ct) genital infections represent a global public health challenge due to their high prevalence and severe reproductive health consequences. These include pelvic inflammatory disease (PID), chronic pelvic pain, infertility, and ectopic pregnancy (1, 2). Infertility and ectopic pregnancy after Ct infection is primarily caused by fibrotic scarring of the oviducts. Additionally, studies have linked Ct with adverse pregnancy outcomes such as stillbirth, infant death, spontaneous abortion, preterm labor, small-for-gestational-age infants, and postpartum endometritis (3). This suggests that Ct may cause lasting endometrial damage.

In the female reproductive tract, immune cells in the vagina and cervix provide the first line of defense against infection, whereas immune cells in the endometrium play a dual role: eliminating pathogens that breach the cervical barrier and maintaining immune tolerance of the embryo during pregnancy. Regulatory T (Treg) cells in the uterus are critical for immune tolerance to foreign fetal antigens(4, 5). Their secretion of anti-inflammatory cytokines such as IL-10 and TGF-β, and expression of inhibitory molecules like CTLA-4 and PD-1, suppress maternal immune responses that could otherwise target the fetus. In contrast, Th17 cells drive proinflammatory responses that can be harmful. The balance between these cell types is crucial for a healthy pregnancy. During Ct infection, the induction of Tregs may hinder the development of effective T cell immunity (6), whereas the activation of chlamydial-specific Th17 cells appears to enhance resistance to reinfection (7). Therefore, while a higher Treg/Th17 ratio supports reproductive health, it may compromise host defense against Ct.

Animal models have also shown that pro-inflammatory signaling and immune cell recruitment are initiated upon Ct infection of host epithelial cells (8, 9). Engagement of pathogen recognition receptors (PRRs) elicits a rapid influx of neutrophils that fail to kill the bacteria while releasing tissue-damaging molecules such as reactive oxygen species and matrix metalloproteases (10–15). Eventually, chlamydia-specific IFN-γ-producing CD4 T cells, activated by dendritic cells, resolve infection. However, danger-associated molecular patterns (DAMPs) released by chlamydiae or dying epithelial cells can perpetuate inflammation through induction of production of TNFα and IL-1α by adjacent epithelium or influxing innate cells, creating a feedforward loop of tissue damage (11, 16, 17). Furthermore, epithelial-mesenchymal transition (EMT)—a dysregulated tissue repair process controlled by microRNAs (miRNAs)—has been implicated in oviduct fibrosis in chlamydia-infected mice (18). During EMT, epithelial cells lose cell-cell contacts and acquire mesenchymal characteristics, including elevated production of extracellular matrix proteins, before transitioning into myofibroblasts.

Mouse models of chlamydial infection demonstrated that the induction of EMT involves TNFα signaling, caspase activation, and cleavage inactivation of Dicer (18, 19) an RNase III enzyme that processes RNA into microRNAs. MicroRNAs (miRNAs) are small non-coding RNAs that play a crucial role in post-transcriptional gene regulation, primarily through translational repression and mRNA degradation. However, miRNAs can also enhance gene expression and translation under certain conditions (20, 21). miRNAs are predicted to control the activity of approximately 30% to 50% of all protein-coding genes. A single miRNA can target multiple mRNAs, while multiple miRNAs can collaborate to finely tune the expression of a single mRNA target (22). Chlamydia infection in mice was associated with reduced expression of miRNAs that normally suppress EMT, fibrosis and tumorigenesis, alongside increased expression of proteins associated with EMT and fibrosis (18, 19)

Human salpingeal tissues are challenging to obtain but minimally invasive endometrial suction catheter biopsies can be collected in an office setting (23). Histological evidence of endometritis correlates with salpingitis observed via laparoscopy (24) and is associated with an increased risk of infertility in women with symptomatic or asymptomatic Ct infection (25). Histologic features resembling endometritis have also been described in surgically removed Fallopian tubes (26), supporting the use of endometrial biopsies for investigating pathogenic mechanisms underlying Ct-induced disease.

In this study, we conducted an integrated analysis of endometrial mRNA and miRNA profiles in cisgender women with high exposure to Ct. Our findings identified an infection-driven mRNA signature that highlights active innate and adaptive immune signaling pathways, along with epithelial-mesenchymal transition (EMT), in women with endometrial Ct infection compared to those with cervical Ct infection only and uninfected women. A parallel miRNA analysis revealed significant downregulation of several miRNAs that typically suppress mRNAs involved in activating TGF-β-related pathways, which drive EMT and regulatory T cell (Treg) differentiation. Additionally, a partially overlapping subset of miRNAs that typically enhance proinflammatory Th17 differentiation was also downregulated. The suppression of these miRNAs during endometrial Ct infection appears to release their regulatory effects, facilitating EMT and increasing the potential for tissue scarring. However, the miRNA-mediated shift in the Treg/Th17 balance, favoring Tregs, may simultaneously act to dampen inflammation.

## Materials and Methods

### Ethics statement.

This study adhered to the Declaration of Helsinki guidelines, and all participants provided written informed consent prior to participation. The study protocols were approved by the Institutional Review Boards for Human Subjects Research at the University of North Carolina and the University of Pittsburgh.

### Study population.

Endometrial biopsy samples were obtained from cisgender female participants enrolled in two independent cohorts with high exposure to Ct infection. Anaerobes and Clearance of Endometritis (ACE) Cohort: Participants were women clinically diagnosed with pelvic inflammatory disease (PID) (27, 28). Diagnostic criteria for enrollment included cervical motion tenderness, uterine tenderness, or adnexal tenderness observed during pelvic examination in sexually active young women experiencing pelvic or lower abdominal pain. T Cell Response Against Chlamydia (TRAC) Cohort: Participants were asymptomatic women identified as being at high risk for Ct infection (29). At enrollment, demographic, behavioral, and medical history data were collected. General physical and pelvic exams were conducted, and blood samples were obtained for immune studies. Participants underwent endometrial biopsy sampling using suction catheters. Additional assessments included Gram-stained vaginal smears for bacterial vaginosis using Nugent scores (30), as well as testing for Ct, *Neisseria gonorrhoeae* (Ng), and *Mycoplasma genitalium* (Mg) in cervical swabs and endometrial biopsies using nucleic acid amplification tests. Histopathological analyses of biopsies were performed (25, 31).

Participants were categorized into three primary groups based on infection status: (1) Endo+: Ct detected in both the endometrium and cervix. Endo+ women were further categorized into subgroups of women with clinical PID (Endo+, PID+), and asymptomatic women (Endo+, PID−). (2) Endo−: Ct detected in the cervix only, without endometrial infection. (3) Uninfected: No Ct infection. For miRNA analyses, women with PID-like symptoms with negative tests for Ct, Ng, and Mg were included as an additional comparison group.

### Study design and workflow.

**Figure 1.**
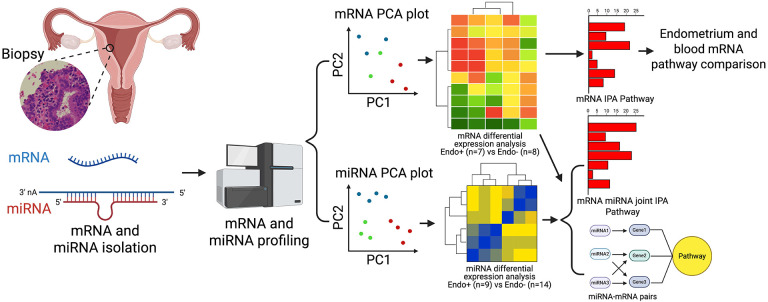
Workflow depicting sample processing, mRNA and miRNA profiling and subsequent analytical methods. Endometrial biopsy samples were processed to profile mRNA from 7 Endo (+) and 8 Endo (−) women, including 4 cervix (+) only and 4 uninfected, and miRNA from 9 Endo (+) and 14 Endo (−) women, including 5 cervix(+) only and 9 uninfected. Relationships among cohort participants based on their transcriptomes were revealed by Principal Component Analysis (PCA). Differentially expressed (DE) mRNAs and miRNAs between Endo+ and Endo− biopsies were determined and functional annotation of DE mRNA and miRNA-mRNA pairs were provided by IPA. Additionally, endometrial biopsy generated mRNA data were compared to previously published blood mRNA data generated from participants in the T Cell Response Against Chlamydia (TRAC) cohort (32).

The study workflow is depicted in Figure 1. Endometrial biopsy samples were analyzed for mRNA and miRNA expression. Principal Component Analysis (PCA) was used to explore transcriptomic relationships among cohort participants. Differentially expressed (DE) mRNAs and miRNAs were identified between Endo+ and Endo−/Uninfected groups. Functional annotations of DE mRNAs and miRNA-mRNA pairs were conducted using Ingenuity Pathway Analysis (IPA). Endometrial mRNA data were also compared with previously published blood mRNA data from the same cohorts (32).

### mRNA and miRNA data collection and processing.

mRNA Extraction and Profiling: Total RNA was extracted from endometrial biopsies stored at −80°C in tubes containing RNA/DNA Shield (Zymo Research, Irvine, CA). After thawing, samples were weighed, minced, and processed for simultaneous DNA and RNA extraction using the Quick DNA/RNA^™^ isolation kit according to the manufacturers protocol (Zymo) with on column DNAse I treatment prior to total RNA elution. Libraries were prepared using the Ovation SoLo RNA-Seq kit (NuGen Technologies) and sequenced on an Illumina HiSeq2500 platform in the High Throughput Sequencing Facility (HTSF) at the University of North Carolina. Gene expression quantification used BBMap (v37.25) and samtools (v1.4.1). Blood mRNA pathway data had been previously generated by microarray hybridization analysis using samples obtained from ACE and TRAC participants (31, 32). miRNA Extraction and Profiling: miRNAs were isolated using HTG EdgeSeq reagents according to the user manuscript, and libraries were synthesized at the HTSF at the University of North Carolina. Barcoded samples were pooled, and sequencing was performed on an Illumina NextSeq 500 platform using a 75-cycle High Output v2 kit. Count data were generated by the EdgeSeq parser software (HTG Molecular Diagnostics, Inc.).

### Data accession.

All endometrial mRNA and miRNA profiles have been deposited in the Gene Expression Omnibus database (https://www.ncbi.nlm.nih.gov/geo/) GEO accession number: GSE290615 for endometrial mRNA, and GSE289941 for endometrial miRNAs. The blood mRNA profiles were retrieved from the previous study (32) with GEO accession number GSE110106.

### Statistical analysis.

Demographic Data: One-way ANOVA was used for numerical variables, while Fisher’s exact test or Chi-square test was applied for categorical variables. RNA-Seq Data: PCA with ComBat-seq (33) correction was applied to address batch effects. mRNAs with expression levels <20 in >50% of samples were excluded, leaving 16,183 mRNAs for analysis. miRNAs with expression levels below mean +3 standard deviations of negative controls were filtered, retaining 253 miRNAs. Normalization was performed using DESeq2 (v1.36.0) (34). PCA of variance by R was used to identify the inherent pattern of samples. Differential Expression Analysis: DE mRNAs and miRNAs were identified using DESeq2 with false discovery rate (FDR) <0.05. All analyses, unless specified, were completed in R (version 4.1.0). Within-sample group clustering heatmaps were generated to visualize DE miRNA expression across groups.

### Functional annotation and pathway enrichment analysis of mRNAs.

Ingenuity Pathway Analysis (IPA) (QIAGEN Inc., https://digitalinsights.qiagen.com/IPA) identified enriched pathways for DE mRNAs, using Fisher’s exact test. Regulatory T cell-related mRNAs not included in IPA were sourced from Gene Ontology (GO) (35) and manually curated from literature.

### Integrated miRNA-mRNA analysis.

Putative target mRNAs of DE miRNAs were identified through IPA’s “miRNA target filter” which incorporates both published experimental and high-confidence computational data. mRNA targets were further filtered to include only those that were also identified as DE mRNAs (FDR<0.05) in this study. We performed a functional annotation of the negatively correlated miRNA-mRNA pairs to identify significantly enriched pathways.

### Systematic review of miRNA functions.

A systematic review identified studies examining DE miRNA functions in human immune-related processes. PubMed searches from 1990 to 2024 used the following search query: all miRNAs AND (infect OR immune OR autoimmune) AND (human OR patient OR healthy donor) AND (“T cell” OR “B cell” OR lymphocyte OR CD4 OR CD8 OR Treg OR Th1 OR Th2 OR Th17 OR Tfh OR neutrophil OR monocyte OR macrophage OR “NK cell” OR “epithelial cell” OR “dendritic cell”) AND (expression OR function OR Differentiation OR activated OR activation OR inhibit OR suppress OR stimulation) NOT (“beta-cell” OR methylation OR stem OR endothelial OR pregnancy). Tumor-related and non-immune studies were excluded, leaving 361 full-text studies curated for miRNA effects on immune and epithelial cell responses including directionality of their effects.

### Comparison of DE mRNA enriched pathways detected in the endometrium versus blood.

We previously identified blood DE mRNAs from women with Ct-induced pelvic inflammatory disease (PID) (Endo+) to those from asymptomatic women with cervical infection only and uninfected women (Endo−/Uninfected) (32). Using Bonferroni-adjusted P values <0.05 as the significance threshold, we compared the significantly enriched pathways for DE mRNAs from this endometrial study and the aforementioned blood study.

## Results

### Characteristics of study participants.

The characteristics of study participants included in the mRNA (N = 15) and miRNA (N = 23) analyses are summarized in Supplementary Tables 1 and 2. Participant group sizes were determined by the quality of mRNA and miRNA isolated from tissue specimens. Most participants were young, African American, unmarried, had some college education, and were insured by Medicaid. A majority reported previous Ct infection, had anti-Ct antibodies, and did not have bacterial vaginosis (BV) by Nugent’s criteria (30) or coinfection with Ng and/or Mg. Demographic variables such as age, race, education, insurance status, and contraceptive use did not differ significantly among groups.

### Distinct gene expression profiles in Endo+ women contrast with minimal impact of cervical Ct infection.

**Figure 2.**
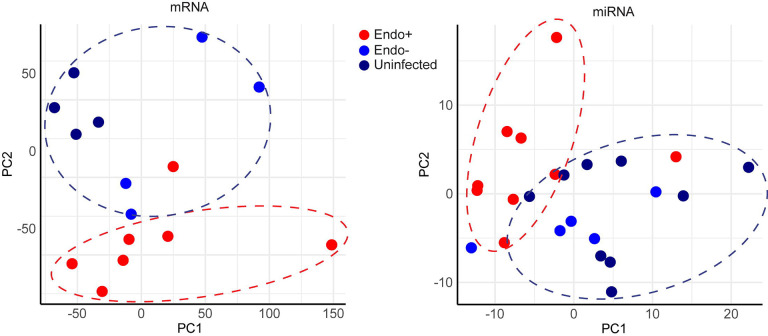
Principal component analysis (PCA) of endometrial transcriptome (A) mRNA and (B) miRNA. Each dot represents one subject, with infection status indicated by color. Red denotes endometrial infection (Endo+), light blue denotes absence of endometrial infection but positive cervical infection (Endo−), and dark blue (Uninfected). The x-axis represents the first principal component, PC1, which accounts for the largest variance of mRNA (A) or miRNA (B) expression, and the y axis, PC2, explains the second largest variance.

Unsupervised principal component analysis (PCA) was performed to assess whether global gene expression profiles were associated with infection status or disease extent. PCA of mRNA data (Figure 2A) showed that most Endo+ women formed a distinct cluster, separate from the Endo− and Uninfected groups, with one outlier in the Endo+ group. Similarly, PCA of miRNA data (Figure 2B) indicated that Endo+ women clustered apart from the Endo− and Uninfected groups. However, PCA did not detect differences between endometrial mRNA or miRNA expression profiles of women with cervical Ct infection or those who were uninfected. The lack of separation between Endo− and Uninfected groups suggests that cervical Ct infection does not significantly impact endometrial gene expression.

### Endometrial inflammation was detected in Endo+ and non-STI PID cases.

**Figure 3.**
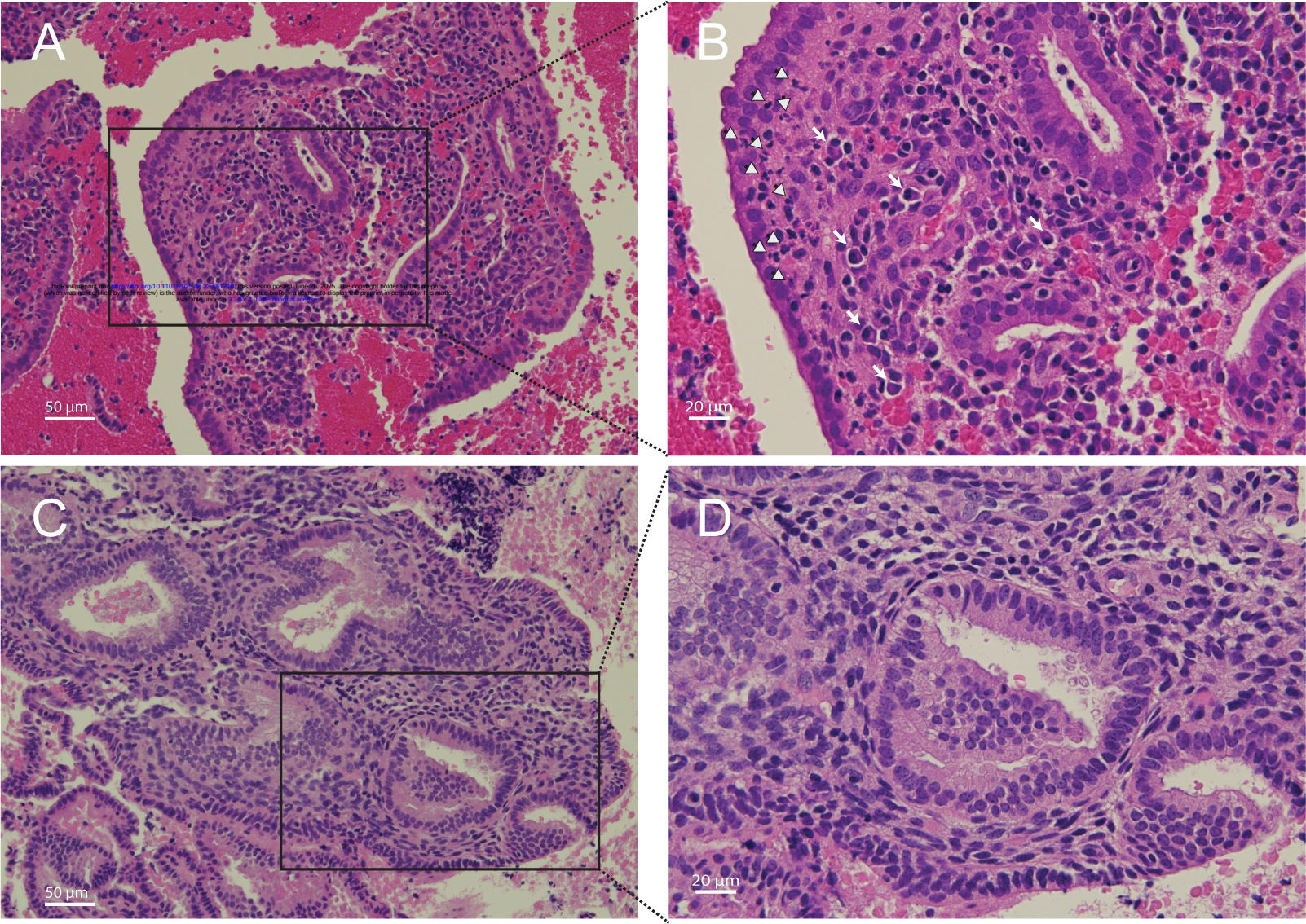
Histopathology of *Chlamydia trachomatis* endometritis. (A, 20X; B, 40X) Histological hematoxylin and eosin-stained endometrial tissue sections from a biopsy taken from a woman positive for *C. trachomatis* in their endometrium. White arrows indicate plasma cells; white arrowheads indicate neutrophils. (C, 20X; D, 40X) Endometrial biopsy section from a woman who was negative for endometrial infection but tested positive for *C. trachomatis* infection at their cervix.

Biopsied tissues from Endo+ women, both symptomatic and asymptomatic, and from women with non-STI-induced PID showed plasma cell and lymphocyte infiltrates in the endometrial stroma. Neutrophils were also observed within the basal lamina of the epithelium and in gland lumens in some Endo+ biopsies. In contrast, 6 evaluable biopsis from women with cervical Ct infection only and 9 evaulable biopsies from uninfected women either lacked inflammatory cells or contained rare mononuclear cells. The remaining biopsies had insufficient tissue for histological evaluation (Figure 3A–D). These pathological data align with the data from PCA analysis indicating clear separation of endometrial transcriptional profiles of Endo+ women from those with Ct infection limited to the cervix.

### Key transcriptional differences in Ct endometritis highlight induction of innate and adaptive immune responses, EMT, and cell cycle dysregulation that is confirmed by functional analysis of miRNA-mRNA interactions.

**Figure 4.**
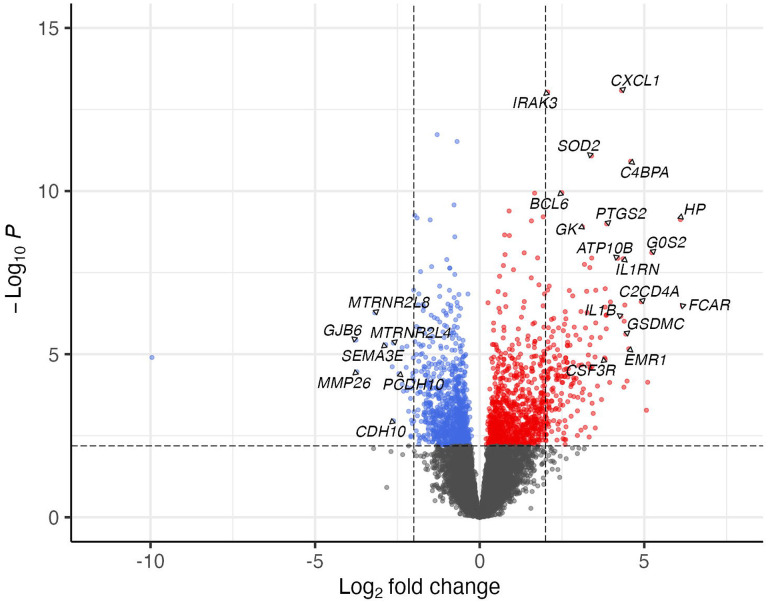
The significance and fold change of DE mRNAs (FDR<0.05) in Endo+ versus Endo−/Uninfected biopsies depicted by volcano plot. Each dot represents one mRNA gene, with red indicating upregulation and blue indicating downregulation in the Endo+ group compared to the Endo−/Uninfected group. Horizontal dashed line indicates FDR=0.05 (−log_10_P=2.2). Left vertical dashed line indicates log2 fold change=−2 (fold change= 0.25) and right vertical dashed line indicates log2 fold change=2 (fold change=4).

Since PCA showed minimal variance between the Endo− and Uninfected groups, their transcriptional data were combined and compared to the Endo+ group. A total of 2,000 mRNAs were significantly DE between Endo+ and Endo−/Uninfected groups (FDR < 0.05), with 1046 upregulated, and 954 downregulated genes in the Endo+ group. The top 100 upregulated and downregulated genes with FDR <0.05 are listed in Supplementary Tables 3 and 4, respectively and displayed in a volcano plot (Figure 4).

Several of the most strongly upregulated genes play crucial roles in local antibody production and germinal center formation. These include transcription factors and coactivators such as *BCL6* and *POU2AF1*, *CXCL13*, a key germinal center chemokine, and antibody Fc receptors including *FCAR*, the IgA receptor, and *FCRL2*, which supports B cell antibody production (Supplementary Table 3). This gene expression pattern was consistent with the marked plasma cell infiltrates observed in women with PID, underscoring the hallmark histology of chronic endometritis (Figure 3A–B) (36–40). Other highly upregulated genes are involved in the acute phase response and cytokine cascade along with secondary mediators, including chemokines, colony-stimulating factors, and prostaglandins, which amplify leukocyte recruitment and local innate immunity (*IL1B*, *IL1RN*, *IRAK3*, *GSDMC*, *C2CD4A*, *CSF3R*, *CXCL1*, *SOD2*, *PTGS2*) (Supplementary Table 3). *PTGS2* which encodes a cyclooxygenase enzyme that catalyzes prostaglandin synthesis and is linked to pain, was up-regulated in Endo+ women, some of whom exhibited PID symptoms.

**Figure 5.**
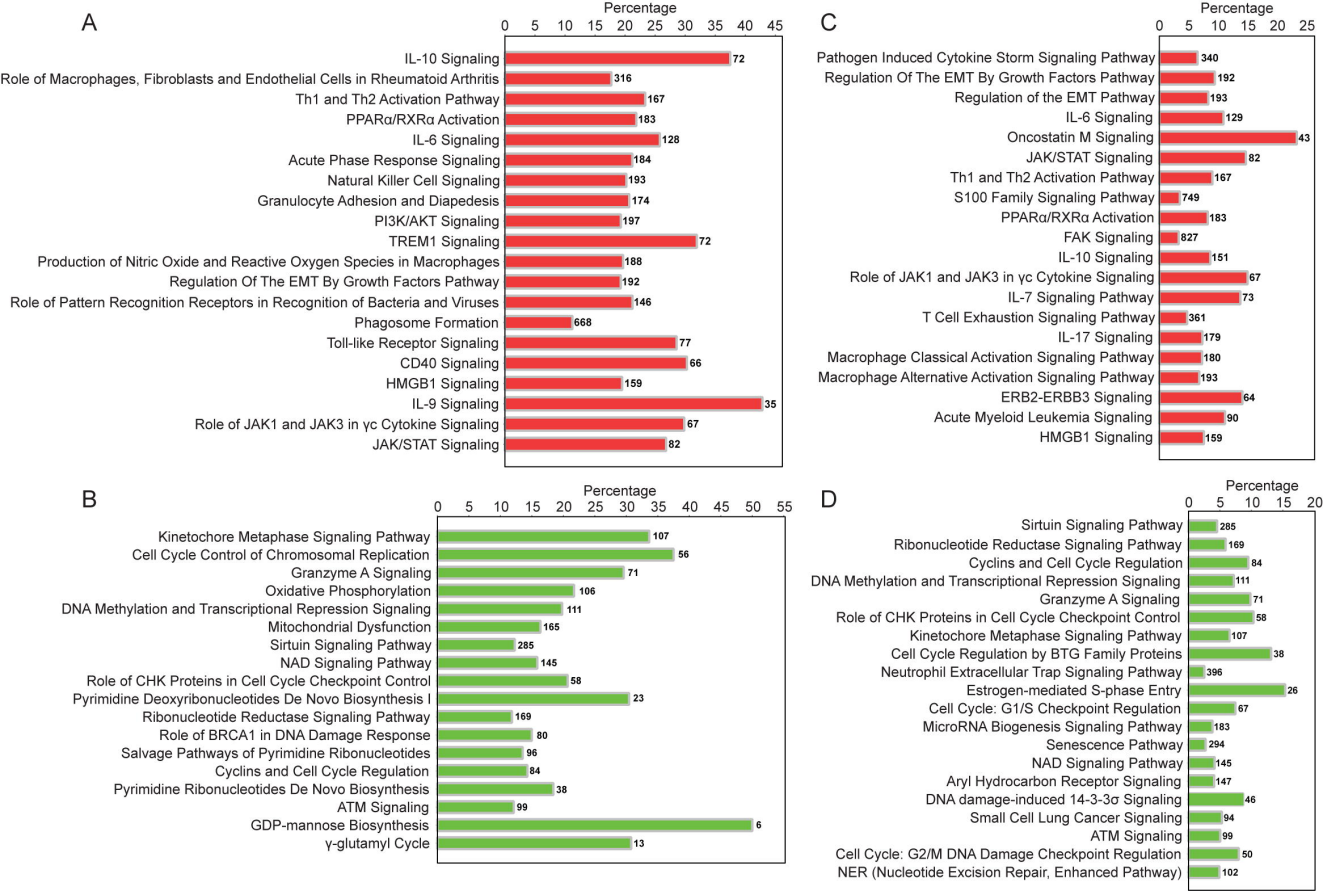
Ingenuity canonical pathways enriched by DE mRNAs and miRNAs in Endo+ compared to Endo−/Uninfected biopsies. (A) Top 20 signaling pathways enriched by significantly upregulated mRNAs in Endo+ biopsies. (B) All 18 pathways enriched by significantly downregulated mRNAs in Endo+ biopsies. (C) Top 20 signaling pathways enriched by downregulated miRNA-upregulated mRNA pairs in Endo+ biopsies. (D) Top 20 signaling pathways enriched by significantly upregulated miRNA-downregulated mRNA pairs in Endo+ biopsies. The percentage is the number of the DE genes present in each pathway, divided by the total number of genes in that pathway according to the IPA database, as listed on the right-hand side of each figure panel.

Upregulated pathways identified by IPA included non-specific inflammatory responses (acute phase response, PPARa/RXRa activation, *IL-6*, *PI3K/AKT*, *JAK/STAT*), innate (*TREM1*, NK cell, nitrous oxide, phagosome formation, Toll-like receptor (TLR) and pattern recognition receptor (PRR) signaling, granulocyte adhesion, and HMGB-1 signaling), and adaptive immune response pathways (*IL-10*, Th1/Th2, CD40 signaling, IL-9 signaling) (Figure 5A, Supplementary Table 5).

IPA also identified EMT pathway enrichment, driven by upregulation of *TGFB2*, *TGFBR2*, *SMAD3*, and *ZEB2* (Figure 5A, Supplementary Table 5). *TGFB2* interacts with its receptor to activate SMAD proteins, including SMAD3. This activation promotes the expression of EMT-related genes such as *ZEB2*, a transcription factor which represses epithelial markers like E-cadherin and enhances mesenchymal markers, playing a pivotal role in EMT progression.

Downregulated genes included membrane adhesion components (*GJB6*, *CDH10*, *PCDH10*) and tissue remodeling enzymes (*MMP26*, *FBN3*, *PLOD1*), as well as genes involved in transcription regulation (*RXRG* and *SPDEF*), protein synthesis (*DHFR*, *GSTZ1*) and modification (*APRT*, *RYCR1*), and cellular metabolism (*KMO*, *NDUFB7*, *UQCRC1*) (Supplementary Table 4). Multiple genes related to histone metabolism were also decreased, potentially altering chromatin structure and impacting gene expression regulation (Figure 4 and Supplementary Tables 4 and 6). Downregulated pathways were enriched in genes involved with cell cycle regulation, mitochondrial dysfunction, and nucleotide biosynthesis (Figure 5B, Supplementary Table 6).

Consistent with the DE mRNA results (Figure 5A), functional enrichment analysis using IPA revealed that downregulated miRNA-mRNA interactions in Endo+ women enriched pathways such as “Regulation of EMT by Growth Factors” (Figure 5C, Supplementary Table 7), highlighting EMT promotion. Additionally, these interactions enriched pathways containing upregulated innate and adaptive immune signaling genes (Figure 5C). In contrast, upregulated miRNAs associated with downregulated mRNAs were enriched in pathways involving cell cycle regulation and DNA damage signaling (Figure 5D), aligning with mRNA findings (Figure 5B and Supplementary Table 8). These results suggest that Ct infection decreases the transcription of miRNAs that normally suppress EMT and immune signaling genes, potentially accelerating scarring and inflammation. Simultaneously, the infection increases the transcription of miRNAs that downregulate mRNAs involved in mucosal epithelial homeostasis, possibly further impairing uterine healing processes.

### Differential expression of miRNAs highlights EMT promotion and Treg/Th17 modulation in endometrial Ct infection.

**Figure 6.**
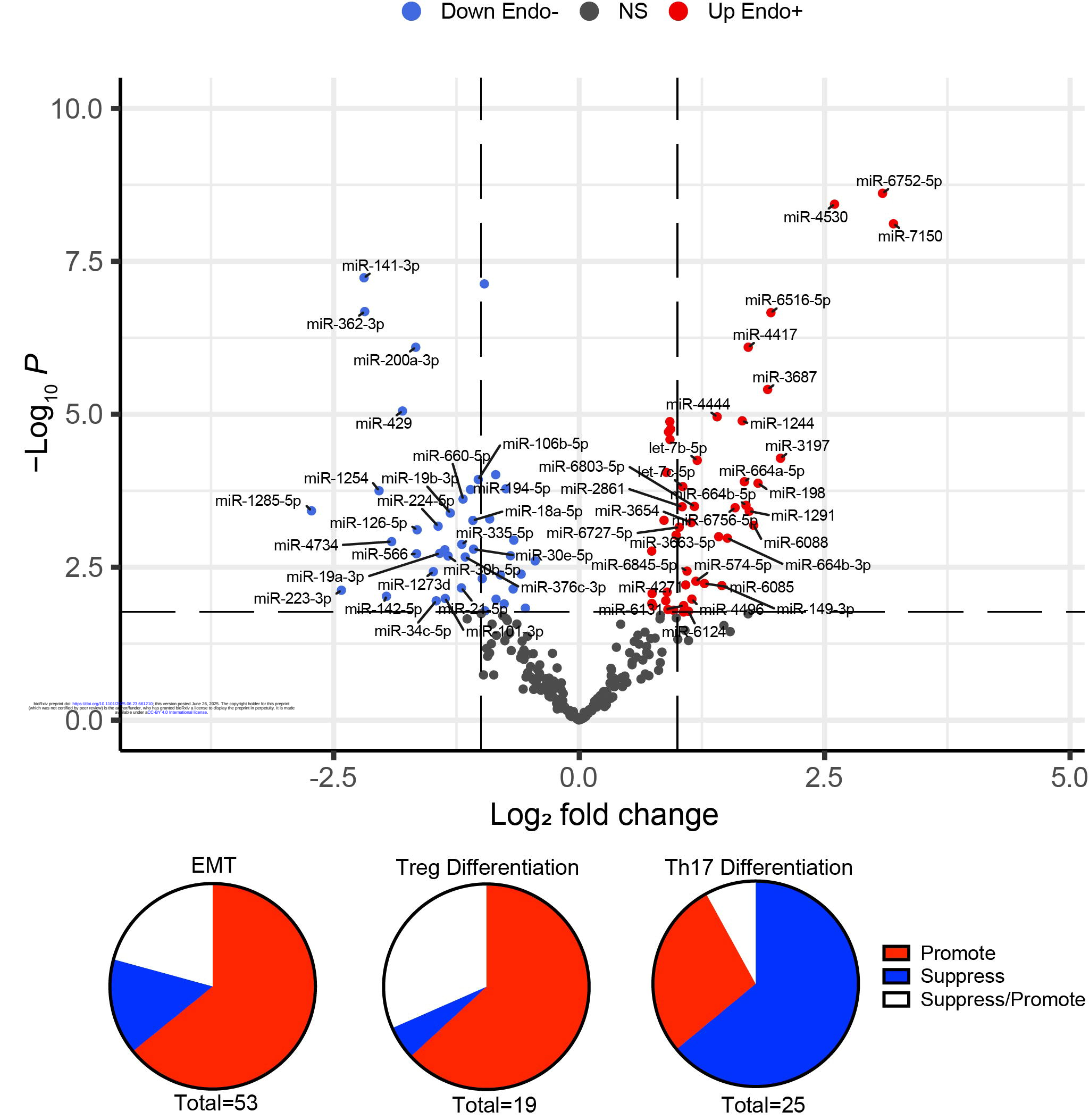
The significance and fold change of DE miRNAs (FDR<0.05) in Endo+ versus Endo−/Uninfected biopsies depicted by (A) volcano plot. Each dot represents one miRNA gene with colors indicating directions of dysregulated expression in the Endo+ group compared to the Endo−/Uninfected group. Horizontal dashed line indicates FDR=0.05 (−log_10_P=2.2). Left vertical dashed line indicates log2 fold change=−1 (fold changes= 0.5) and right vertical dashed line indicates log2 fold change=1 (fold change=2). All significant DE miRNAs (FDR<0.05) with fold changes>2 or <0.5 are highlighted. **DE miRNAs linked to (B) EMT, (C) Tregs, and (D) Th17 differentiation are depicted by pie charts.** Red, blue and white colors indicate overall enhancing, suppressing, and enhancing/suppressing effects of miRNAs, respectively, in Endo+ women compared to Endo−/Uninfected women.

A total of 89 miRNAs were significantly DE (FDR<0.05) between Endo+ and Endo− women. Among these, 47 miRNAs were upregulated, and 42 were downregulated (Supplementary Tables 9 and 10). miRNAs with fold changes >2 and <1/2 are depicted in Figure 6A. The majority of (53 of 89) DE miRNAs were associated with EMT with 32 miRNAs that normally dampen EMT being downregulated and 4 that increase EMT being upregulated (Figure 6B), leading to an overall effect of enhancing EMT (Table 1).

IPA’s target filter and literature review identified target mRNAs linked to TGF-β signaling, crucial for EMT induction. These included *JAK1/STAT3* (103), Smad-dependent and (104) independent *TGF-β* pathways, and the autocrine signaling network for EMT involving *TGF-β* and *ZEB2* (104). Transcription factors (e.g., *ZEB2*, *FOXO1*) and pathways like PI3K-AKT were upregulated, emphasizing EMT promotion. Among the 25 most significantly downregulated miRNAs in Endo+ samples, seven (miR-141–3p, miR-200a-3p, miR-106b-5p, miR-224–5p, miR-20a-5p, miR-21–5p, and miR-223–3p) were identified through literature review as directly suppressing *TGF-β* production. Consequently, their downregulation in Endo+ women likely enhances *TGF-β* activity, which may suppress chronic inflammation but also promote EMT, potentially contributing to adverse pathological outcomes.

Among the 89 differentially expressed (DE) miRNAs, 32 were associated with T cell regulation, including subclasses such as Tregs, Th17, and Th1. *TGF-β* plays a key role in the differentiation of both Tregs and Th17 cells. However, the presence of additional cytokines, particularly interleukin-6 (*IL-6*), shifts differentiation toward Th17 cells. Specifically, low *TGF-β* concentrations combined with *IL-6* promote Th17 differentiation, whereas high *TGF-β* levels suppress *IL-6* signaling and favor Treg differentiation (105).

Twenty miRNAs were linked to Treg differentiation, with 12 downregulated miRNAs typically functioning to suppress Tregs (Figure 6C, Table 2). Notably, miR-146a-5p, which promotes Tregs, was upregulated, while miR-148a-3p, another Treg-enhancing miRNA, was reduced. These expression patterns suggest an overall increase in Treg differentiation in Endo+ women.

For Th17 differentiation, 25 miRNAs were identified (Figure 6D, Table 3). Of these, 14 miRNAs that typically enhance Th17 cells were significantly reduced, 10 of which also function to suppress Tregs. Additionally, two miRNAs that normally inhibit Th17 differentiation were upregulated. Conversely, six miRNAs that suppress Th17 differentiation were downregulated, and miR-149–3p, which promotes Th17 cells, was increased. Overall, 16 of the 25 DE miRNAs are predicted to reduce Th17 differentiation, while 7 are associated with increased Th17 differentiation.

Several target mRNAs were linked to promoting Treg differentiation over Th17 cells. These include transcription factors such as *FOXO1*, *RUNX1*, *IRF1*, and *BATF*, as well as key *TGF-β* pathway genes (*TGFBR2*, *SMAD3*, *STAT5*, *STAT3*, and *IL-10R*). Collectively, these findings indicate a shift toward increased Treg activity and reduced Th17 differentiation. This shift may help limit tissue-damaging neutrophil responses but could come at the cost of impaired Th17/Th1-mediated protection against reinfection (7).

**Figure 7.**
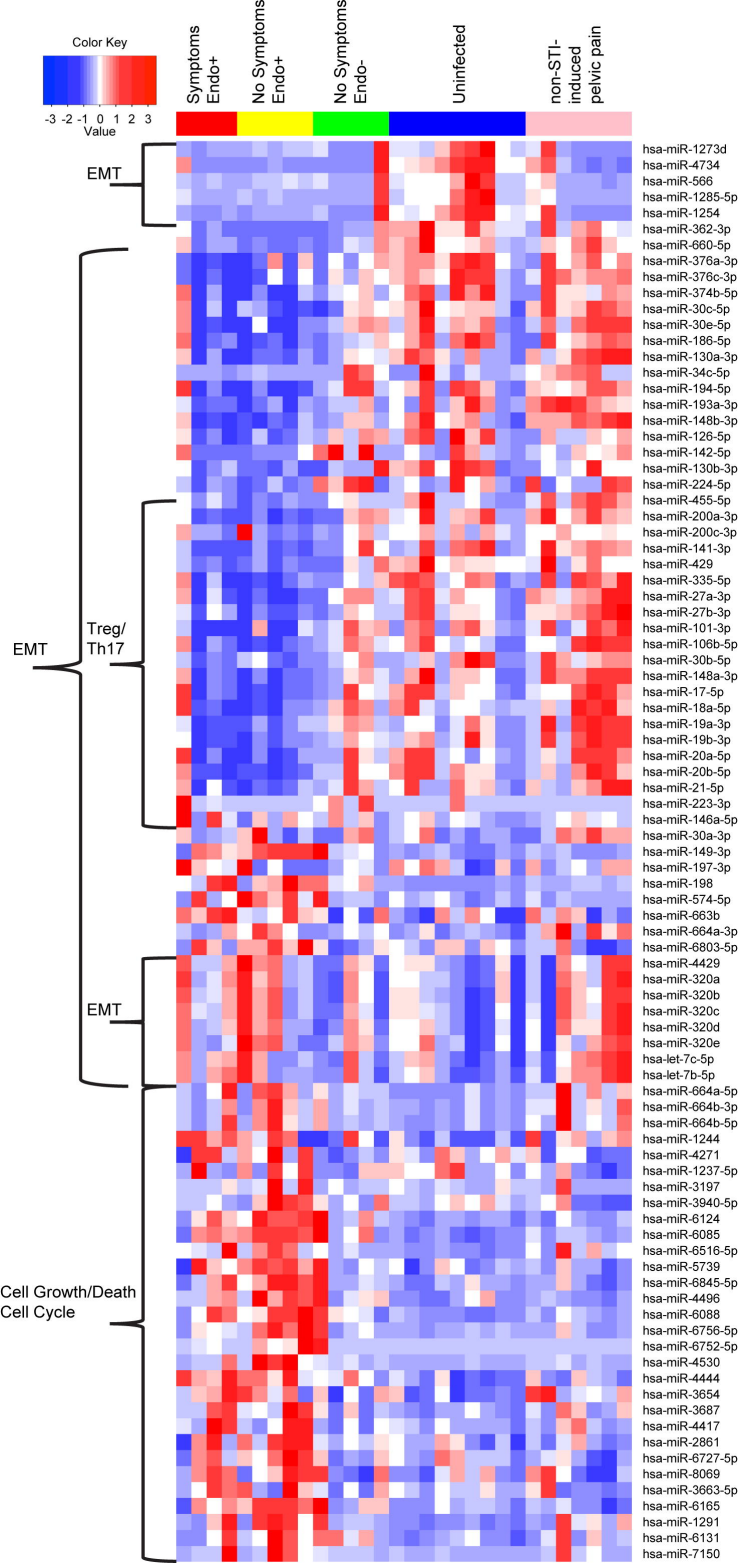
A within-sample group hierarchical clustering heatmap of DE miRNAs revealed distinct expression patterns across groups. Heatmap columns indicate individual patients with red: Endo+ with pelvic pain; yellow: Endo+ without symptoms; green: Endo− without symptoms; blue: uninfected without symptoms; pink: pelvic pain without sexually transmitted infection (STI). Heatmap rows indicate genes. Blue: low expression miRNAs, white: intermediately expressed miRNAs, and red: highly expressed miRNAs.

A within-sample group clustering heatmap of DE miRNAs (Figure 7) revealed distinct expression patterns across groups: Endo+ women with and without symptoms, Endo−, Uninfected, and women with non-STI-induced pelvic pain. One cluster of miRNAs was downregulated in Endo+ women (symptomatic and asymptomatic) compared to Endo−, Uninfected, and women with non-STI-induced pelvic pain. Literature review suggests these miRNAs typically suppress EMT (Table 1), implying their downregulation in Endo+ women may promote EMT, regardless of symptom presence. Notably, this downregulation was not observed in non-STI pelvic pain cases, suggesting a link to active Ct infection (Figure 7).

However, a minor cluster of miRNAs (mir-1273, mir-1285–5p, mir-1254, mir-4734, and mir-566) that normally dampen mRNAs promoting EMT (Table 1, Figure 7), was downregulated in Endo+, 4 of 5 Endo−, and 5 of 7 non-STI pelvic pain cases with recent prior Ct infection, indicating potential enhancement of EMT processes irrespective of current Ct ascension or symptom status. Each of these miRNAs have been studied in various cancer types, and reportedly have both positive (miR-1273 (137), miR-4734) and negative roles (miR-1285–5p (138), miR-1254 (139), miR-566 (140)) in promoting cell proliferation, invasion, and metastasis. Furthermore, four miRNAs (miR-320b (102, 141), let-7c-5p and let-7b-5p (79)) reported to induce EMT and promote oncogenesis were upregulated in Endo+ women and those with non-STI-induced pelvic pain (Table 1, Figure 7).

### Distinct immune and fibrosis-related pathways were detected in endometrial vs. blood mRNA profiles in women with chlamydial endometritis.

In a previous study (32), we compared blood-derived mRNA profiles from women with Ct-induced pelvic inflammatory disease (PID) (Endo+) to those from asymptomatic women with cervical infection only and uninfected women (Endo−/Uninfected). A comparison of the top enriched pathways identified in blood with those found in the endometrium of Endo+ women revealed notable differences (Table 4). Shared pathways related to innate immune responses, including granulocyte adhesion, *TREM1* signaling, Toll-like receptor (TLR) signaling, integrin signaling, *IL-1*, *IL-10*, and *IL-6* response pathways, as well as *JAK/STAT* signaling. Endometrium--specific responses included natural killer (NK) cell signaling pathways, *IL-23*, *STAT3*, and Th17 activation pathways which can lead to chronic inflammation if excessively activated (142), multiple adaptive T cell response pathways (Th1/Th17, Th2, and *IL-7*), and fibrosis-related pathways, *TGF-β* signaling and EMT. These sharply contrasted with the blood profiles of Endo+ women, where TCR signaling pathways—including mTOR, which integrates immune signals and metabolic cues for T cell maintenance and activation, and *ICOS*-*ICOSL* signaling which delivers the co-stimulatory signals that promote T cell activation and differentiation—were significantly downregulated, and activation of EMT pathways was undetected. Conversely, type I interferon signaling pathways were prominent in blood-borne profiles, but displayed minimal enrichment in the endometrium.

## Discussion

The findings from this study improve understanding of how Ct infection modulates the molecular landscape of the human endometrium, emphasizing the interplay between immune responses, EMT, and regulatory miRNA activity. These results provide insight into how Ct infection may contribute to adverse pregnancy outcomes, including stillbirth and preterm labor, as well as the Ct-induced complication of chronic pelvic pain.

The integrated transcriptomic and miRNA analyses revealed distinct molecular signatures in the endometrial tissues of women with endometrial Ct infection (Endo+), highlighting active innate and adaptive immune signaling pathways alongside EMT promotion. The transcriptional patterns of upregulated genes associated with the acute phase response as well as genes that promote antibody-producing cells and cell-mediated immunity correlate with histological findings of endometritis, marked by a predominance of plasma cell infiltrates mixed with subepithelial neutrophils and lymphocytes. Other upregulated genes in Endo+ women, such as *TGFB2*, *TGFBR2*, and *SMAD3*, collectively drive EMT through Smad-dependent and independent mechanisms, while transcription factors like *ZEB2* suppress epithelial markers to facilitate mesenchymal transitions. The significant downregulation of EMT-suppressing miRNAs, including miR-141–3p and miR-200a-3p, further supports this shift toward EMT, aligning with prior observations in murine models (18, 19, 143). These molecular alterations are consistent with enhanced tissue remodeling, fibrosis, and scarring—hallmarks of the Ct-induced complications of tubal factor infertility and ectopic pregnancy. The additional detection of upregulated miRNAs that depress gene pathways engaged in nucleotide synthesis and cell cycle control indicate a further mechanism whereby miRNA dysregulation could inhibit healing of infected endometrial tissue. While this study focused on endometrial biopsy tissues, it is highly likely that similar mechanisms are at work in the delicate oviduct. EMT and fibrosis in the oviduct could impair critical reproductive processes, including gamete transport, fertilization, early embryo development, immune defense, and structural integrity—all of which are essential for successful reproduction.

The enrichment of *TGF-β* signaling and its downstream transcription factors, such as *ZEB2*, in Endo+ samples underscores the role of this pathway in driving EMT. Downregulated miRNAs that typically suppress *TGF-β* signaling, including miR-141–3p and miR-200a-3p, likely amplify this effect. These molecular changes are consistent with previous studies in mouse models, which demonstrated that dysregulated EMT contributes to fibrosis and oviductal scarring. Igietseme et al. (18) showed that *Chlamydia muridarum*-induced TNFα signaling and caspase activation were linked to the downregulation of miRNAs that typically inhibit EMT and fibrosis. Similarly, alterations in *TGF-β*, *TNF*, *ZEB*, and the miR-200 family observed in Endo+ women were also identified in infertile mice, with multiple differentially expressed miRNAs involved in EMT overlapping between the two studies (143, 144) demonstrated significant downregulation of miRNAs that suppress fibrosis, including members of the miR-200 family in mice with oviduct pathology following *C. muridarum* infection. This overlap in miRNA dysregulation between human and mouse studies suggests a conserved *Chlamydia*-induced EMT mechanism across species. The downregulation of these miRNAs, combined with the activation of *TGF-β* signaling, likely creates a microenvironment conducive to tissue remodeling and scarring. This process may represent a critical step in the progression from infection to tubal factor infertility.

A notable finding was the miRNA-driven shift in the Treg/Th17 balance. Among the 89 DE miRNAs, many were linked to immune cell regulation, particularly promoting Treg differentiation and function. The downregulation of miRNAs that typically suppress Tregs (e.g., miR-200a-3p, miR-374b-5p) suggests an enhanced Treg response, potentially favoring immune tolerance at the expense of effective Th1/Th17-mediated immunity. This interpretation is bolstered by the upregulation of miR-146a-5p, known to support Treg function, and the increased expression of Treg-associated mRNAs, such as TGFBR2 and Smad3.

Conversely, the downregulation of Th17-promoting miRNAs, such as miR-21–5p, miR-19b-3p, and miR-223–3p, indicates a reduction in Th17 activity. Since Th17 cells enhance neutrophil recruitment, decreased Th17 activity could mitigate tissue-damaging neutrophil responses, key drivers of chlamydial disease pathogenesis (15). However, given Ct’s intracellular developmental cycle, and its production of Chlamydial Protease Activation Factor (CPAF), which impairs neutrophil function (145), neutrophils have limited efficacy in eliminating Ct, and reduced neutrophil activation may have little impact on infection resolution. However, Th17 cells exhibit plasticity, transitioning between Th17 and Th1 phenotypes, and have been strongly associated with resistance to chlamydial reinfection (7). Thus, the observed shift in the Treg/Th17 balance has contrasting implications. Enhanced Treg/Th17 activity may reduce chronic inflammation and preserve endometrial function; while simultaenously, impeding development of protective immunity, potentially contributing to the high rates of reinfection observed in some cohort participants (7). This delicate trade-off underscores the challenges of therapeutically targeting these pathways, as interventions could disrupt the balance between immune suppression and effective defense.

Comparative analyses between endometrial and previously published blood mRNA profiles highlight tissue-specific responses to Ct infection. While blood profiles revealed activation of interferon-mediated signaling and downregulation of adaptive T cell pathwys, endometrial responses were dominated by activation of T cells (e.g., Treg, Th1, Th17), EMT, and *TGF-β*-driven immune regulation. These differences underscore the localized nature of Ct-induced endometrial inflammation and fibrosis, which may not be readily detectable through systemic biomarkers. This localized EMT induction aligns with the histological evidence of fibrosis observed in Ct-affected Fallopian tubes, suggesting a direct link between Ct gene regulation in the upper genital tract and long-term reproductive sequelae. These findings underscore the importance of localized studies for understanding the distinct molecular landscape of Ct-infected reproductive tissues.

We acknowledge several limitations of this study. The sample size for the endometrial transcriptome was constrained by the difficulty of obtaining sufficient high-quality endometrial tissue for mRNA and miRNA profiling. Additionally, the cross-sectional design of the study limits our ability to assess the temporal progression of infection and associated pathology. Future studies with larger sample sizes and longitudinal designs are needed to validate these findings and provide a more comprehensive understanding into the progression of Ct-induced pathology and the persistence of molecular changes post-infection. Extending these analyses to other sexually transmitted infections could determine whether similar miRNA-mediated mechanisms drive reproductive tract inflammation and scarring across different pathogens.

Despite these limitations, our study provides a new and comprehensive view of molecular changes in the endometrium during Ct infection, highlighting critical pathways that drive tissue remodeling, fibrosis, and immune dysregulation. The findings also raise concerns about asymptomatic Endo+ women, who exhibited similar molecular profiles to symptomatic individuals, suggesting that subclinical endometrial inflammation may contribute to reproductive morbidity, which supports prior studies indicating that asymptomatic women with Ct-induced endometritis have an increased risk for infertility (25).

In summary, this study demonstrates that endometrial Ct infection induces a miRNA-mediated regulatory network that promotes EMT and alters immune cell balance, favoring Treg differentiation over Th17 responses. These molecular changes likely contribute to tissue scarring and impaired immune protection, offering new insights into the pathogenesis of Ct-induced reproductive morbidity. Targeting these pathways may provide a foundation for innovative therapeutic strategies to mitigate the long-term consequences of Ct infection.

## Supplementary Material

Supplement 1

## Figures and Tables

**Table 1.** DE miRNAs in Endo+ compared to Endo− associated with EMT.

DE miRNAs	Fold Change	P value	FDR	Expression changes in Endo+ compared to Endo-	Function of miRNAs	Overall effects	Citations
miR-566	0.32	0.002	0.009	Decrease (−)	↓EMT	**Promote EMT**	(41)
miR-200a-3p	0.32	<0.001	<0.001	Decrease (−)	↓EMT		(42)
miR-200c-3p	0.59	0.013	0.039	Decrease (−)	↓EMT		(42)
miR-141–3p	0.22	<0.001	<0.001	Decrease (−)	↓EMT		(43)
miR-429	0.29	<0.001	<0.001	Decrease (−)	↓EMT		
miR-376a-3p	0.51	0.016	0.048	Decrease (−)	↓EMT		(44)
miR-376c-3p	0.45	0.002	0.009	Decrease (−)	↓EMT		(45)
miR-374b-5p	0.39	0.002	0.008	Decrease (−)	↓EMT		(46)
miR-30b-5p	0.4	0.002	0.009	Decrease (−)	↓EMT		(47)
miR-30c-5p	0.68	0.015	0.045	Decrease (−)	↓EMT		(47)
miR-30e-5p	0.47	0.002	0.008	Decrease (−)	↓EMT		(47)
miR-186–5p	0.62	0.002	0.009	Decrease (−)	↓EMT		(48)
miR-130a-3p	0.63	0.001	0.006	Decrease (−)	↓EMT		(49)
miR-335–5p	0.44	0.001	0.007	Decrease (−)	↓EMT		(50)
miR-17–5p	0.6	<0.001	0.002	Decrease (−)	↓EMT		(51)
miR-18a-5p	0.47	0.001	0.004	Decrease (−)	↓EMT		(51)
miR-19a-3p	0.37	0.002	0.009	Decrease (−)	↓EMT		(51)
miR-19b-3p	0.4	<0.001	0.003	Decrease (−)	↓EMT		(51)
miR-20a-5p	0.5	0.005	0.019	Decrease (−)	↓EMT		(51)
miR-20b-5p	0.51	<0.001	<0.001	Decrease (−)	↓EMT		(52, 53)
miR-34c-5p	0.36	0.011	0.036	Decrease (−)	↓EMT		(54)
miR-101–3p	0.39	0.01	0.035	Decrease (−)	↓EMT		(55)
miR-194–5p	0.46	<0.001	0.002	Decrease (−)	↓EMT		(56)
miR-193a-3p	0.57	0.004	0.017	Decrease (−)	↓EMT		(57)
miR-148a-3p	0.53	0.001	0.004	Decrease (−)	↓EMT		(58, 59)
mir-148b-3p	0.73	0.002	0.011	Decrease (−)	↓EMT		(60)
miR-126–5p	0.32	<0.001	0.004	Decrease (−)	↓EMT		(61, 62)
miR-142–5p	0.26	0.009	0.032	Decrease (−)	↓EMT		(63)
miR-1285–5p	0.15	<0.001	0.003	Decrease (−)	↓EMT		(64)
mir-1254	0.24	<0.001	0.002	Decrease (−)	↓EMT		(65)
miR-6803–5p	2.26	<0.001	0.003	Increase (+)	↑EMT		(66)
miR-664a-3p	1.82	0.001	0.004	Increase (+)	↑EMT		(67)
miR-663b	1.98	0.001	0.005	Increase (+)	↑EMT		(68)
miR-197–3p	1.67	0.012	0.039	Increase (+)	↑EMT		(69)
miR-106b-5p	0.49	<0.001	0.001	Decrease (−)	↑EMT	Suppress EMT	(70–72)
miR-198	3.54	<0.001	0.001	Increase (+)	↓EMT		(73)
mir-30a-3p	1.67	0.008	0.027	Increase (+)	↓EMT		(74)
miR-320a	1.9	<0.001	<0.001	Increase (+)	↓EMT		(75)
miR-320c	1.88	<0.001	<0.001	Increase (+)	↓EMT		(76)
miR-320d	1.85	<0.001	0.001	Increase (+)	↓EMT		(77)
let-7c-5p	2.08	<0.001	0.002	Increase (+)	↓EMT		(78)
miR-4429	1.9	<0.001	<0.001	Increase (+)	↓EMT		(79, 80)
miR-130b-3p	0.63	0.007	0.026	Decrease (−)	↓↑EMT	Suppress/Promote EMT	(81–83)
miR-21–5p	0.6	0.024	0.05	Decrease (−)	↓↑EMT		(84, 85)
miR-27a-3p	0.55	<0.001	0.001	Decrease (−)	↓↑EMT		(86, 87)
miR-27b-3p	0.66	0.004	0.017	Decrease (−)	↓↑EMT		(88, 89)
miR-224–5p	0.37	0.001	0.004	Decrease (−)	↓↑EMT		(90, 91)
miR-455–5p	0.56	0.011	0.035	Decrease (−)	↓↑EMT		(92, 93)
miR-223–3p	0.19	0.008	0.027	Decrease (−)	↓↑EMT		(94)
miR-149–3p	2.74	0.006	0.024	Increase (+)	↓↑EMT		(95, 96)
miR-574–5p	2.28	0.005	0.021	Increase (+)	↓↑EMT		(97, 98)
miR-146a-5p	2.09	0.017	0.048	Increase (+)	↓↑EMT		(99, 100)
miR-320b	1.91	<0.001	<0.001	Increase (+)	↓↑EMT		(75, 101)

**Table 3.** DE miRNAs in Endo+ compared to Endo−, associated with Th17 cell differentiation.

DE miRNAs	Fold change	P value	FDR	Expression changes in Endo+ compared to Endo-	Function of miRNAs	Overall effects	Citations
miR-200a-3p	0.32	8.02E-07	2.25E-05	Reduced (−)	↑ Th17	Suppress Th17 differentiation	(105, 106)
miR-141-3p	0.22	5.87E-08	3.71E-06	Reduced (−)	↑ Th17		(108)
miR-200c-3p	0.59	1.26E-02	3.93E-02	Reduced (−)	↑ Th17		(105)
miR-429	0.29	8.84E-06	2.03E-04	Reduced (−)	↑ Th17		
miR-374b-5p	0.39	1.64E-03	7.98E-03	Reduced (−)	↑ Th17		(123)
miR-106b-5p	0.49	1.17E-04	1.34E-03	Reduced (−)	↑ Th17		(113)
miR-130b-3p	0.63	7.17E-03	2.59E-02	Reduced (−)	↑ Th17		(124)
miR-17	0.60	1.65E-04	1.59E-03	Reduced (−)	↑ Th17		(125)
miR-18a	0.47	5.44E-04	3.53E-03	Reduced (−)	↑ Th17		(125)
miR-19a	0.37	1.88E-03	8.76E-03	Reduced (−)	↑ Th17		(125)
miR-19b	0.4	4.12E-04	2.89E-03	Reduced (−)	↑ Th17		(125)
miR-20a	0.50	4.86E-03	1.92E-02	Reduced (−)	↑ Th17		(125)
mir-30c-5p	0.68	1.48E-02	4.45E-02	Reduced (−)	↑ Th17		(126)
mir-223-3p	0.19	7.53E-03	2.68E-02	Reduced (−)	↑ Th17		(114, 127)
Let-7b	2.3	5.66E-05	7.54E-04	Increased (+)	↓ Th17		(128)
Let-7c	2.1	1.52E-04	1.54E-03	Increased (+)	↓ Th17		(128)
miR-30a-3p	1.7	8.51E-03	2.95E-02	Reduced (−)	↓ Th17	Promote Th17 differentiation	(129)
miR-20a	0.50	4.86E-03	1.92E-02	Reduced (−)	↓ Th17		(125)
miR-20b	0.51	7.39E-08	3.74E-06	Reduced (−)	↓ Th17		(130)
miR-21-5p	0.44	6.90E-03	2.53E-02	Reduced (−)	↓ Th17		(122, 126)
miR-148a	0.53	5.15E-04	3.52E-03	Reduced (−)	↓ Th17		(131) (125)
miR-148b	0.73	2.50E-03	1.07E-02	Reduced (−)	↓ Th17		(132)
miR-149-3p	2.7	6.32E-03	2.35E-02	Increased (+)	↑ Th17		(133)
miR-27 a	0.55	9.72E-05	1.17E-03	Reduced (−)	↓↑ Th17	Suppress/promote Th17 differentiation	(134, 135)
miR-27 b	0.66	4.10E-03	1.67E-02	Reduced (−)	↓↑Th17		(134, 135)

**Table 4.** Comparison of pathways enriched by significantly differentially expressed (DE) mRNAs in Endo+ versus Endo− women, as determined from either endometrial biopsy or blood samples#.

IPA Pathway	Endometrium		Blood	
	Regulation direction in Endo+	Adjusted P*	Regulation direction in Blood	Adjusted P**
**Up-regulated in Endometrium and Blood**				
IL-10 Signaling	↑	3.83E-15	↑	1.29E-06
IL-6 Signaling	↑	3.83E-13	↑	1.32E-05
Granulocyte Adhesion and Diapedesis	↑	3.04E-11	↑	6.92E-03
TREM1 Signaling	↑	6.07E-11	↑	7.78E-09
Production of Nitric Oxide and Reactive				
Oxygen Species in Macrophages	↑	6.07E-11	↑	6.15E-04
PI3K/AKT Signaling	↑	6.07E-11	↑	1.48E-03
Phagosome Formation	↑	1.92E-09	↑	1.66E-04
Toll-like Receptor Signaling	↑	2.42E-09	↑	3.47E-05
IL-8 Signaling	↑	9.84E-08	↑	5.13E-07
IL-3 Signaling	↑	1.71E-06	↑	3.47E-02
IL-17 Signaling	↑	1.30E-05	↑	4.16E-02
Leukocyte Extravasation Signaling	↑	4.20E-05	↑	1.86E-04
IL-1 Signaling	↑	5.29E-03	↑	1.38E-02
Dendritic Cell Maturation	↑	1.63E-02	↑	2.57E-05
Integrin Signaling	↑	1.81E-02	↑	9.34E-04
T Helper Cell Differentiation	↑	2.05E-02	↑	1.02E-03
Interferon Signaling	↑	3.88E-02	↑	1.95E-12
**Up-regulated Only in Endometrium**				
Th1 and Th2 Activation Pathway	↑	3.04E-14		##NS
Natural Killer Cell Signaling	↑	6.07E-12		NS
Regulation Of The Epithelial Mesenchymal				
Transition	↑	1.21E-10		NS
IL-23 Signaling Pathway	↑	7.47E-08		NS
IL-7 Signaling Pathway	↑	4.10E-07		NS
STAT3 Pathway	↑	4.50E-07		NS
TGF-β Signaling	↑	1.67E-06		NS
IL-13 Signaling Pathway	↑	3.83E-03		NS
T Cell Exhaustion Signaling Pathway	↑	2.47E-02		NS
Th17 Activation Pathway	↑	3.74E-02		NS
**Up-regulated in Endometrium and Down regulated in Blood**				
T Cell Receptor Signaling	↑	5.29E-03	↓	8.52E-03
ICOS-ICOSL Signaling in T Helper Cells	↑	2.36E-02	↓	7.78E-04
mTOR Signaling	↑	NS	↓	4.90E-12
Regulation of eIF4 and p70S6K Signaling	↑	NS	↓	3.89E-11

# Data from reference (32).

* Bonferroni adjusted P values in endometrium: P*482 for endometrium (482 total pathways).

** Bonferroni adjusted P values in blood: P*389 for blood (389 total pathways).

##NS: Not Significant.

Light gray indicates pathways that were enriched by significantly upregulated genes only in endometrial biopsy specimens.

Dark gray indicates pathways that were enriched by significantly down-regulated genes only in blood specimens.

## References

[1] Chow J. M., Yonekura M. L., Richwald G. A., Greenland S., Sweet R. L., and Schachter J.. 1990. The association between Chlamydia trachomatis and ectopic pregnancy. A matched-pair, case-control study. JAMA 263: 3164–3167. 2348526

[2] Passos L. G., Terraciano P., Wolf N., Oliveira F. D. S., Almeida I., and Passos E. P.. 2022. The Correlation between Chlamydia Trachomatis and Female Infertility: A Systematic Review. Rev Bras Ginecol Obstet 44: 614–620. 35576969 10.1055/s-0042-1748023 PMC9948125

[3] Tang W., Mao J., Li K. T., Walker J. S., Chou R., Fu R., Chen W., Darville T., Klausner J., and Tucker J. D.. 2020. Pregnancy and fertility-related adverse outcomes associated with Chlamydia trachomatis infection: a global systematic review and meta-analysis. Sex Transm Infect 96: 322–329. 31836678 10.1136/sextrans-2019-053999 PMC7292777

[4] Huang N., Chi H., and Qiao J.. 2020. Role of Regulatory T Cells in Regulating Fetal-Maternal Immune Tolerance in Healthy Pregnancies and Reproductive Diseases. Front Immunol 11: 1023. 32676072 10.3389/fimmu.2020.01023 PMC7333773

[5] La Rocca C., Carbone F., Longobardi S., and Matarese G.. 2014. The immunology of pregnancy: regulatory T cells control maternal immune tolerance toward the fetus. Immunol Lett 162: 41–48. 24996040 10.1016/j.imlet.2014.06.013

[6] Stary G., Olive A., Radovic-Moreno A. F., Gondek D., Alvarez D., Basto P. A., Perro M., Vrbanac V. D., Tager A. M., Shi J., Yethon J. A., Farokhzad O. C., Langer R., Starnbach M. N., and von Andrian U. H.. 2015. VACCINES. A mucosal vaccine against Chlamydia trachomatis generates two waves of protective memory T cells. Science 348: aaa8205. 26089520 10.1126/science.aaa8205 PMC4605428

[7] Yount K. S., Chen C. J., Kollipara A., Liu C., Mokashi N. V., Zheng X., Bagwell C. B., Poston T. B., Wiesenfeld H. C., Hillier S. L., O’Connell C. M., Stanley N., and Darville T.. 2024. Unique T cell signatures associated with reduced Chlamydia trachomatis reinfection in a highly exposed cohort. bioRxiv. 10.1172/jci.insight.189388 PMC11991011 40014387

[8] Darville T., and Hiltke T. J.. 2010. Pathogenesis of genital tract disease due to Chlamydia trachomatis. J Infect Dis 201 Suppl 2: S114–125. 20524234 10.1086/652397 PMC3150527

[9] Rank R. G., and Whittum-Hudson J. A.. 2010. Protective immunity to chlamydial genital infection: evidence from animal studies. J Infect Dis 201 Suppl 2: S168–177. 20470052 10.1086/652399

[10] Hart K. M., Murphy A. J., Barrett K. T., Wira C. R., Guyre P. M., and Pioli P. A.. 2009. Functional expression of pattern recognition receptors in tissues of the human female reproductive tract. J Reprod Immunol 80: 33–40. 19406482 10.1016/j.jri.2008.12.004 PMC2744441

[11] Darville T., O’Neill J. M., Andrews C. W. Jr., Nagarajan U. M., Stahl L., and Ojcius D. M.. 2003. Toll-like receptor-2, but not Toll-like receptor-4, is essential for development of oviduct pathology in chlamydial genital tract infection. J Immunol 171: 6187–6197. 14634135 10.4049/jimmunol.171.11.6187

[12] Frazer L. C., O’Connell C. M., Andrews C. W. Jr., Zurenski M. A., and Darville T.. 2011. Enhanced neutrophil longevity and recruitment contribute to the severity of oviduct pathology during Chlamydia muridarum infection. Infect Immun 79: 4029–4041. 21825059 10.1128/IAI.05535-11 PMC3187238

[13] Lee H. Y., Schripsema J. H., Sigar I. M., Murray C. M., Lacy S. R., and Ramsey K. H.. 2010. A link between neutrophils and chronic disease manifestations of Chlamydia muridarum urogenital infection of mice. FEMS Immunol Med Microbiol 59: 108–116. 20370824 10.1111/j.1574-695X.2010.00668.x

[14] Rank R. G., Whittimore J., Bowlin A. K., Dessus-Babus S., and Wyrick P. B.. 2008. Chlamydiae and polymorphonuclear leukocytes: unlikely allies in the spread of chlamydial infection. FEMS Immunol Med Microbiol 54: 104–113. 18657107 10.1111/j.1574-695X.2008.00459.x PMC2925246

[15] Ramsey K. H., Sigar I. M., Schripsema J. H., Shaba N., and Cohoon K. P.. 2005. Expression of matrix metalloproteinases subsequent to urogenital Chlamydia muridarum infection of mice. Infect Immun 73: 6962–6973. 16177376 10.1128/IAI.73.10.6962-6973.2005 PMC1230927

[16] Murthy A. K., Li W., Chaganty B. K., Kamalakaran S., Guentzel M. N., Seshu J., Forsthuber T. G., Zhong G., and Arulanandam B. P.. 2011. Tumor necrosis factor alpha production from CD8+ T cells mediates oviduct pathological sequelae following primary genital Chlamydia muridarum infection. Infect Immun 79: 2928–2935. 21536799 10.1128/IAI.05022-11 PMC3191981

[17] Gyorke C. E., Kollipara A., Allen J. t., Zhang Y., Ezzell J. A., Darville T., Montgomery S. A., and Nagarajan U. M.. 2020. IL-1alpha Is Essential for Oviduct Pathology during Genital Chlamydial Infection in Mice. J Immunol 205: 3037–3049. 33087404 10.4049/jimmunol.2000600

[18] Igietseme J. U., Omosun Y., Stuchlik O., Reed M. S., Partin J., He Q., Joseph K., Ellerson D., Bollweg B., George Z., Eko F. O., Bandea C., Liu H., Yang G., Shieh W. J., Pohl J., Karem K., and Black C. M.. 2015. Role of Epithelial-Mesenchyme Transition in Chlamydia Pathogenesis. PLoS One 10: e0145198. 26681200 10.1371/journal.pone.0145198 PMC4683008

[19] Igietseme J. U., Omosun Y., Nagy T., Stuchlik O., Reed M. S., He Q., Partin J., Joseph K., Ellerson D., George Z., Goldstein J., Eko F. O., Bandea C., Pohl J., and Black C. M.. 2018. Molecular Pathogenesis of Chlamydia Disease Complications: Epithelial-Mesenchymal Transition and Fibrosis. Infect Immun 86. 10.1128/IAI.00585-17 PMC5736829 29084894

[20] Filipowicz W., Bhattacharyya S. N., and Sonenberg N.. 2008. Mechanisms of post-transcriptional regulation by microRNAs: are the answers in sight? Nat Rev Genet 9: 102–114. 18197166 10.1038/nrg2290

[21] O’Brien J., Hayder H., Zayed Y., and Peng C.. 2018. Overview of MicroRNA Biogenesis, Mechanisms of Actions, and Circulation. Front Endocrinol (Lausanne) 9: 402. 30123182 10.3389/fendo.2018.00402 PMC6085463

[22] Lewis B. P., Shih I. H., Jones-Rhoades M. W., Bartel D. P., and Burge C. B.. 2003. Prediction of mammalian microRNA targets. Cell 115: 787–798. 14697198 10.1016/s0092-8674(03)01018-3

[23] Dacco M. D., Moustafa M., Papoutsis D., Georgantzis D., Halmos G., and Magos A.. 2012. An audit of using the H Pipelle for endometrial sampling at outpatient hysteroscopy and literature review comparison with the Pipelle de Cornier. Eur J Obstet Gynecol Reprod Biol 165: 299–301. 22884589 10.1016/j.ejogrb.2012.07.021

[24] Kiviat N. B., Wolner-Hanssen P., Eschenbach D. A., Wasserheit J. N., Paavonen J. A., Bell T. A., Critchlow C. W., Stamm W. E., Moore D. E., and Holmes K. K.. 1990. Endometrial histopathology in patients with culture-proved upper genital tract infection and laparoscopically diagnosed acute salpingitis. Am J Surg Pathol 14: 167–175. 2137304 10.1097/00000478-199002000-00008

[25] Wiesenfeld H. C., Hillier S. L., Meyn L. A., Amortegui A. J., and Sweet R. L.. 2012. Subclinical pelvic inflammatory disease and infertility. Obstet Gynecol 120: 37–43. 22678036 10.1097/AOG.0b013e31825a6bc9

[26] Hunt J. L., and Lynn A. A.. 2002. Histologic features of surgically removed fallopian tubes. Arch Pathol Lab Med 126: 951–955. 12171494 10.5858/2002-126-0951-HFOSRT

[27] Wiesenfeld H. C., Meyn L. A., Darville T., Macio I. S., and Hillier S. L.. 2021. A Randomized Controlled Trial of Ceftriaxone and Doxycycline, With or Without Metronidazole, for the Treatment of Acute Pelvic Inflammatory Disease. Clin Infect Dis 72: 1181–1189. 32052831 10.1093/cid/ciaa101 PMC8028096

[28] Workowski K. A., Bolan G. A., C. Centers for Disease, and Prevention. 2015. Sexually transmitted diseases treatment guidelines, 2015. MMWR Recomm Rep 64: 1–137. PMC5885289 26042815

[29] Russell A. N., Zheng X., O’Connell C. M., Taylor B. D., Wiesenfeld H. C., Hillier S. L., Zhong W., and Darville T.. 2016. Analysis of Factors Driving Incident and Ascending Infection and the Role of Serum Antibody in Chlamydia trachomatis Genital Tract Infection. J Infect Dis 213: 523–531. 26347571 10.1093/infdis/jiv438 PMC4721908

[30] Nugent R. P., Krohn M. A., and Hillier S. L.. 1991. Reliability of diagnosing bacterial vaginosis is improved by a standardized method of gram stain interpretation. J Clin Microbiol 29: 297–301. 1706728 10.1128/jcm.29.2.297-301.1991 PMC269757

[31] Wiesenfeld H. C., Sweet R. L., Ness R. B., Krohn M. A., Amortegui A. J., and Hillier S. L.. 2005. Comparison of acute and subclinical pelvic inflammatory disease. Sex Transm Dis 32: 400–405. 15976596 10.1097/01.olq.0000154508.26532.6a

[32] Zheng X., O’Connell C. M., Zhong W., Nagarajan U. M., Tripathy M., Lee D., Russell A. N., Wiesenfeld H., Hillier S., and Darville T.. 2018. Discovery of Blood Transcriptional Endotypes in Women with Pelvic Inflammatory Disease. J Immunol 200: 2941–2956. 29531169 10.4049/jimmunol.1701658 PMC5893373

[33] Zhang Y., Parmigiani G., and Johnson W. E.. 2020. ComBat-seq: batch effect adjustment for RNA-seq count data. NAR Genom Bioinform 2: lqaa078. 33015620 10.1093/nargab/lqaa078 PMC7518324

[34] Love M. I., Huber W., and Anders S.. 2014. Moderated estimation of fold change and dispersion for RNA-seq data with DESeq2. Genome Biol 15: 550. 25516281 10.1186/s13059-014-0550-8 PMC4302049

[35] Gene Ontology C., Aleksander S. A., Balhoff J., Carbon S., Cherry J. M., Drabkin H. J., Ebert D., Feuermann M., Gaudet P., Harris N. L., Hill D. P., Lee R., Mi H., Moxon S., Mungall C. J., Muruganugan A., Mushayahama T., Sternberg P. W., Thomas P. D., …, and Westerfield M.. 2023. The Gene Ontology knowledgebase in 2023. Genetics 224.

[36] Rangel-Moreno J., Moyron-Quiroz J., Kusser K., Hartson L., Nakano H., and Randall T. D.. 2005. Role of CXC chemokine ligand 13, CC chemokine ligand (CCL) 19, and CCL21 in the organization and function of nasal-associated lymphoid tissue. J Immunol 175: 4904–4913. 16210592 10.4049/jimmunol.175.8.4904

[37] Carlsen H. S., Baekkevold E. S., Johansen F. E., Haraldsen G., and Brandtzaeg P.. 2002. B cell attracting chemokine 1 (CXCL13) and its receptor CXCR5 are expressed in normal and aberrant gut associated lymphoid tissue. Gut 51: 364–371. 12171958 10.1136/gut.51.3.364 PMC1773345

[38] Tolnay M. 2022. Lymphocytes sense antibodies through human FCRL proteins: Emerging roles in mucosal immunity. J Leukoc Biol 111: 477–487. 33884658 10.1002/JLB.4RU0221-102RR

[39] King H. W., Wells K. L., Shipony Z., Kathiria A. S., Wagar L. E., Lareau C., Orban N., Capasso R., Davis M. M., Steinmetz L. M., James L. K., and Greenleaf W. J.. 2021. Integrated single-cell transcriptomics and epigenomics reveals strong germinal center-associated etiology of autoimmune risk loci. Sci Immunol 6: eabh3768. 34623901 10.1126/sciimmunol.abh3768 PMC8859880

[40] Wang H., Jain S., Li P., Lin J. X., Oh J., Qi C., Gao Y., Sun J., Sakai T., Naghashfar Z., Abbasi S., Kovalchuk A. L., Bolland S., Nutt S. L., Leonard W. J., and Morse H. C. 3rd. 2019. Transcription factors IRF8 and PU.1 are required for follicular B cell development and BCL6-driven germinal center responses. Proc Natl Acad Sci U S A 116: 9511–9520. 31000603 10.1073/pnas.1901258116 PMC6511064

[41] Di Ruocco F., Basso V., Rivoire M., Mehlen P., Ambati J., De Falco S., and Tarallo V.. 2018. Alu RNA accumulation induces epithelial-to-mesenchymal transition by modulating miR-566 and is associated with cancer progression. Oncogene 37: 627–637. 28991230 10.1038/onc.2017.369 PMC5799714

[42] Feng J., Hu S., Liu K., Sun G., and Zhang Y.. 2022. The Role of MicroRNA in the Regulation of Tumor Epithelial-Mesenchymal Transition. Cells 11. 10.3390/cells11131981 PMC9265548 35805066

[43] Wang S., Zhang M., Zhang T., Deng J., Xia X., and Fang X.. 2020. microRNA-141 inhibits TGF-beta1-induced epithelial-to-mesenchymal transition through inhibition of the TGF-beta1/SMAD2 signalling pathway in endometriosis. Arch Gynecol Obstet 301: 707–714. 31903498 10.1007/s00404-019-05429-w PMC7060956

[44] Sun Y., Shen S., Liu X., Tang H., Wang Z., Yu Z., Li X., and Wu M.. 2014. MiR-429 inhibits cells growth and invasion and regulates EMT-related marker genes by targeting Onecut2 in colorectal carcinoma. Mol Cell Biochem 390: 19–30. 24402783 10.1007/s11010-013-1950-x PMC3972435

[45] Zhang L., Chen Y., Wang H., Zheng X., Li C., and Han Z.. 2018. miR-376a inhibits breast cancer cell progression by targeting neuropilin-1 NR. Onco Targets Ther 11: 5293–5302. 30214235 10.2147/OTT.S173416 PMC6124787

[46] Lin S., Tan L., Luo D., Peng X., Zhu Y., and Li H.. 2019. Linc01278 inhibits the development of papillary thyroid carcinoma by regulating miR-376c-3p/DNM3 axis. Cancer Manag Res 11: 8557–8569. 31572010 10.2147/CMAR.S217886 PMC6756842

[47] Zhao X., Zhang X., Zhang X., Jiang T., Zhai J., Wang H., Huang M., Lang R., and He Q.. 2021. MiR-374b-5p inhibits KDM5B-induced epithelial-mesenchymal transition in pancreatic cancer. Am J Cancer Res 11: 3907–3920. 34522457 PMC8414380

[48] Song K., Jiang Y., Zhao Y., Xie Y., Zhou J., Yu W., and Wang Q.. 2020. Members of the miR-30 family inhibit the epithelial-to-mesenchymal transition of non-small-cell lung cancer cells by suppressing XB130 expression levels. Oncol Lett 20: 68. 32863901 10.3892/ol.2020.11929 PMC7436119

[49] Lu J., Zhao Z., and Ma Y.. 2020. miR-186 Represses Proliferation, Migration, Invasion, and EMT of Hepatocellular Carcinoma via Directly Targeting CDK6. Oncol Res 28: 509–518. 32698940 10.3727/096504020X15954139263808 PMC7751224

[50] Tian X., Fei Q., Du M., Zhu H., Ye J., Qian L., Lu Z., Zhang W., Wang Y., Peng F., Chen J., Liu B., Li Q., He X., and Yin L.. 2019. miR-130a-3p regulated TGF-beta1-induced epithelial-mesenchymal transition depends on SMAD4 in EC-1 cells. Cancer Med 8: 1197–1208. 30741461 10.1002/cam4.1981 PMC6434193

[51] Du W., Tang H., Lei Z., Zhu J., Zeng Y., Liu Z., and Huang J. A.. 2019. miR-335–5p inhibits TGF-beta1-induced epithelial-mesenchymal transition in non-small cell lung cancer via ROCK1. Respir Res 20: 225. 31638991 10.1186/s12931-019-1184-x PMC6805547

[52] Fang L. L., Wang X. H., Sun B. F., Zhang X. D., Zhu X. H., Yu Z. J., and Luo H.. 2017. Expression, regulation and mechanism of action of the miR-17–92 cluster in tumor cells (Review). Int J Mol Med 40: 1624–1630. 29039606 10.3892/ijmm.2017.3164 PMC5716450

[53] Qi J. C., Yang Z., Zhang Y. P., Lu B. S., Yin Y. W., Liu K. L., Xue W. Y., Qu C. B., and Li W.. 2019. miR-20b-5p, TGFBR2, and E2F1 Form a Regulatory Loop to Participate in Epithelial to Mesenchymal Transition in Prostate Cancer. Front Oncol 9: 1535. 32010624 10.3389/fonc.2019.01535 PMC6974577

[54] Khalilian S., Abedinlou H., Hussen B. M., Imani S. Z. H., and Ghafouri-Fard S.. 2022. The emerging role of miR-20b in human cancer and other disorders: Pathophysiology and therapeutic implications. Front Oncol 12: 985457. 36582800 10.3389/fonc.2022.985457 PMC9792503

[55] Morizane R., Fujii S., Monkawa T., Hiratsuka K., Yamaguchi S., Homma K., and Itoh H.. 2014. miR-34c attenuates epithelial-mesenchymal transition and kidney fibrosis with ureteral obstruction. Sci Rep 4: 4578. 24694752 10.1038/srep04578 PMC3974136

[56] Li C., Xia J., Yao W., Yang G., Tian Y., Qi Y., and Hao C.. 2022. Mechanism of LncRNA XIST/ miR-101–3p/ZEB1 axis in EMT associated with silicosis. Toxicol Lett 360: 11–19. 35271943 10.1016/j.toxlet.2022.03.001

[57] Dong P., Kaneuchi M., Watari H., Hamada J., Sudo S., Ju J., and Sakuragi N.. 2011. MicroRNA-194 inhibits epithelial to mesenchymal transition of endometrial cancer cells by targeting oncogene BMI-1. Mol Cancer 10: 99. 21851624 10.1186/1476-4598-10-99 PMC3173388

[58] Chen J., Gao S., Wang C., Wang Z., Zhang H., Huang K., Zhou B., Li H., Yu Z., Wu J., and Chen C.. 2016. Pathologically decreased expression of miR-193a contributes to metastasis by targeting WT1-E-cadherin axis in non-small cell lung cancers. J Exp Clin Cancer Res 35: 173. 27821145 10.1186/s13046-016-0450-8 PMC5100283

[59] Li J., Song Y., Wang Y., Luo J., and Yu W.. 2013. MicroRNA-148a suppresses epithelial-to-mesenchymal transition by targeting ROCK1 in non-small cell lung cancer cells. Mol Cell Biochem 380: 277–282. 23670799 10.1007/s11010-013-1682-y

[60] Hu B., Chen Z., Wang X., Chen F., Song Z., and Cao C.. 2021. MicroRNA-148a-3p Directly Targets SERPINE1 to Suppress EMT-Mediated Colon Adenocarcinoma Progression. Cancer Manag Res 13: 6349–6362. 34408494 10.2147/CMAR.S302777 PMC8364830

[61] Miscianinov V., Martello A., Rose L., Parish E., Cathcart B., Mitic T., Gray G. A., Meloni M., Al Haj Zen A., and Caporali A.. 2018. MicroRNA-148b Targets the TGF-beta Pathway to Regulate Angiogenesis and Endothelial-to-Mesenchymal Transition during Skin Wound Healing. Mol Ther 26: 1996–2007. 29843955 10.1016/j.ymthe.2018.05.002 PMC6094488

[62] Jia Z., Zhang Y., Xu Q., Guo W., and Guo A.. 2018. miR-126 suppresses epithelial-to-mesenchymal transition and metastasis by targeting PI3K/AKT/Snail signaling of lung cancer cells. Oncol Lett 15: 7369–7375. 29725450 10.3892/ol.2018.8207 PMC5920360

[63] Jiang R., Zhang C., Liu G., Gu R., and Wu H.. 2017. MicroRNA-126 Inhibits Proliferation, Migration, Invasion, and EMT in Osteosarcoma by Targeting ZEB1. J Cell Biochem 118: 3765–3774. 28379605 10.1002/jcb.26024

[64] Yao Y., Xu Q., Yan L., Jiao Y., Su Q., Li X., Liu C., and Zhao F.. 2020. MiRNA-128 and MiRNA-142 Regulate Tumorigenesis and EMT in Oral Squamous Cell Carcinoma Through HOXA10. Cancer Manag Res 12: 9987–9997. 33116855 10.2147/CMAR.S250093 PMC7567577

[65] Pao S. I., Lin L. T., Chen Y. H., Chen C. L., and Chen J. T.. 2021. Repression of Smad4 by MicroRNA-1285 moderates TGF-beta-induced epithelial-mesenchymal transition in proliferative vitreoretinopathy. PLoS One 16: e0254873. 34383767 10.1371/journal.pone.0254873 PMC8360606

[66] Jiang M., Shi L., Yang C., Ge Y., Lin L., Fan H., He Y., Zhang D., Miao Y., and Yang L.. 2019. miR-1254 inhibits cell proliferation, migration, and invasion by down-regulating Smurf1 in gastric cancer. Cell Death Dis 10: 32. 30631050 10.1038/s41419-018-1262-x PMC6328618

[67] Yan S., Cheng M., Duan Q., Wang Z., Gao W., Ren B., and Xu D.. 2019. MiR-6803–5p Promotes Cancer Cell Proliferation and Invasion via PTPRO/NF-kappaB Axis in Colorectal Cancer. Mediators Inflamm 2019: 8128501. 31827380 10.1155/2019/8128501 PMC6886338

[68] Zhu X., Ju S., Yuan F., Chen G., Shu Y., Li C., Xu Y., Luo J., and Xia L.. 2017. microRNA-664 enhances proliferation, migration and invasion of lung cancer cells. Exp Ther Med 13: 3555–3562. 28588679 10.3892/etm.2017.4433 PMC5450804

[69] You X., Wang Y., Meng J., Han S., Liu L., Sun Y., Zhang J., Sun S., Li X., Sun W., Dong Y., and Zhang Y.. 2021. Exosomal miR-663b exposed to TGF-beta1 promotes cervical cancer metastasis and epithelial-mesenchymal transition by targeting MGAT3. Oncol Rep 45. 10.3892/or.2021.7963 PMC7877003 33649791

[70] Hamada S., Satoh K., Miura S., Hirota M., Kanno A., Masamune A., Kikuta K., Kume K., Unno J., Egawa S., Motoi F., Unno M., and Shimosegawa T.. 2013. miR-197 induces epithelial-mesenchymal transition in pancreatic cancer cells by targeting p120 catenin. J Cell Physiol 228: 1255–1263. 23139153 10.1002/jcp.24280

[71] Miao L. J., Yan S., Zhuang Q. F., Mao Q. Y., Xue D., He X. Z., and Chen J. P.. 2019. miR-106b promotes proliferation and invasion by targeting Capicua through MAPK signaling in renal carcinoma cancer. Onco Targets Ther 12: 3595–3607. 31190862 10.2147/OTT.S184674 PMC6525582

[72] Luan W., Ding Y., Xi H., Ruan H., Lu F., Ma S., and Wang J.. 2021. Exosomal miR-106b-5p derived from melanoma cell promotes primary melanocytes epithelial-mesenchymal transition through targeting EphA4. J Exp Clin Cancer Res 40: 107. 33741023 10.1186/s13046-021-01906-w PMC7980627

[73] Yang C., Dou R., Wei C., Liu K., Shi D., Zhang C., Liu Q., Wang S., and Xiong B.. 2021. Tumor-derived exosomal microRNA-106b-5p activates EMT-cancer cell and M2-subtype TAM interaction to facilitate CRC metastasis. Mol Ther 29: 2088–2107. 33571679 10.1016/j.ymthe.2021.02.006 PMC8178444

[74] Wang S., Zhang X., Yang C., and Xu S.. 2019. MicroRNA-198–5p inhibits the migration and invasion of non-small lung cancer cells by targeting fucosyltransferase 8. Clin Exp Pharmacol Physiol 46: 955–967. 31381176 10.1111/1440-1681.13154

[75] Wang H., Kanmangne D., Li R., Qian Z., Xia X., Wang X., and Wang T.. 2020. miR-30a-3p suppresses the proliferation and migration of lung adenocarcinoma cells by downregulating CNPY2. Oncol Rep 43: 646–654. 31894275 10.3892/or.2019.7423

[76] Wang X., Wang J., Huang G., Li Y., and Guo S.. 2021. miR-320a-3P alleviates the epithelial-mesenchymal transition of A549 cells by activation of STAT3/SMAD3 signaling in a pulmonary fibrosis model. Mol Med Rep 23. 10.3892/mmr.2021.11996 PMC7974326 33760151

[77] Liu X., Song J., Kang Y., Wang Y., and Chen A.. 2022. CircPDSS1 promotes the proliferation, invasion, migration, and EMT of breast cancer cell via regulating miR-320c/CKAP5 axis. Cancer Cell Int 22: 238. 35902921 10.1186/s12935-022-02657-0 PMC9331068

[78] Shi S., Hu X., Xu J., Liu H., and Zou L.. 2018. MiR-320d suppresses the progression of breast cancer via lncRNA HNF1A-AS1 regulation and SOX4 inhibition. RSC Adv 8: 19196–19207. 35539662 10.1039/c8ra01200h PMC9080600

[79] Hou B., Ishinaga H., Midorikawa K., Nakamura S., Hiraku Y., Oikawa S., Ma N., Takeuchi K., and Murata M.. 2018. Let-7c inhibits migration and epithelial-mesenchymal transition in head and neck squamous cell carcinoma by targeting IGF1R and HMGA2. Oncotarget 9: 8927–8940. 29507664 10.18632/oncotarget.23826 PMC5823619

[80] Liang L., Zheng Y. W., and Wang Y. L.. 2020. miR-4429 Regulates the Proliferation, Migration, Invasion, and Epithelial-Mesenchymal Transition of Cervical Cancer by Targeting FOXM1. Cancer Manag Res 12: 5301–5312. 32669877 10.2147/CMAR.S244167 PMC7338043

[81] Pan H., Hong Y., Yu B., Li L., and Zhang X.. 2019. miR-4429 Inhibits Tumor Progression and Epithelial-Mesenchymal Transition Via Targeting CDK6 in Clear Cell Renal Cell Carcinoma. Cancer Biother Radiopharm 34: 334–341. 30844301 10.1089/cbr.2018.2697

[82] Dong P., Karaayvaz M., Jia N., Kaneuchi M., Hamada J., Watari H., Sudo S., Ju J., and Sakuragi N.. 2013. Mutant p53 gain-of-function induces epithelial-mesenchymal transition through modulation of the miR-130b-ZEB1 axis. Oncogene 32: 3286–3295. 22847613 10.1038/onc.2012.334 PMC3705163

[83] Li B. L., Lu C., Lu W., Yang T. T., Qu J., Hong X., and Wan X. P.. 2013. miR-130b is an EMT-related microRNA that targets DICER1 for aggression in endometrial cancer. Med Oncol 30: 484. 23392577 10.1007/s12032-013-0484-0

[84] Chang R. M., Xu J. F., Fang F., Yang H., and Yang L. Y.. 2016. MicroRNA-130b promotes proliferation and EMT-induced metastasis via PTEN/p-AKT/HIF-1alpha signaling. Tumour Biol 37: 10609–10619. 26861561 10.1007/s13277-016-4919-z

[85] Arisan E. D., Rencuzogullari O., Cieza-Borrella C., Miralles Arenas F., Dwek M., Lange S., and Uysal-Onganer P.. 2021. MiR-21 Is Required for the Epithelial-Mesenchymal Transition in MDA-MB-231 Breast Cancer Cells. Int J Mol Sci 22. 10.3390/ijms22041557 PMC7913884 33557112

[86] Ghosh A., Ranjan N., Jiang L., Ansari A. H., Degyatoreva N., Ahluwalia S., Arya D. P., and Maiti S.. 2022. Fine-tuning miR-21 expression and inhibition of EMT in breast cancer cells using aromatic-neomycin derivatives. Mol Ther Nucleic Acids 27: 685–698. 35070496 10.1016/j.omtn.2021.12.027 PMC8763640

[87] Zhao N., Sun H., Sun B., Zhu D., Zhao X., Wang Y., Gu Q., Dong X., Liu F., Zhang Y., and Li X.. 2016. miR-27a-3p suppresses tumor metastasis and VM by down-regulating VE-cadherin expression and inhibiting EMT: an essential role for Twist-1 in HCC. Sci Rep 6: 23091. 26980408 10.1038/srep23091 PMC4793289

[88] Pan G., Liu Y., Shang L., Zhou F., and Yang S.. 2021. EMT-associated microRNAs and their roles in cancer stemness and drug resistance. Cancer Commun (Lond) 41: 199–217. 33506604 10.1002/cac2.12138 PMC7968884

[89] Zhang J., Hua X., Qi N., Han G., Yu J., Yu Y., Wei X., Li H., Chen X., Leng C., Liu Q., Lu Y., and Li Y.. 2020. MiR-27b suppresses epithelial-mesenchymal transition and chemoresistance in lung cancer by targeting Snail1. Life Sci 254: 117238. 31887300 10.1016/j.lfs.2019.117238

[90] Suzuki H. I., Katsura A., Mihira H., Horie M., Saito A., and Miyazono K.. 2017. Regulation of TGF-beta-mediated endothelial-mesenchymal transition by microRNA-27. J Biochem 161: 417–420. 28338957 10.1093/jb/mvx017 PMC5412016

[91] Lu Y., Huang W., Chen H., Wei H., Luo A., Xia G., Deng X., and Zhang G.. 2019. MicroRNA-224, negatively regulated by c-jun, inhibits growth and epithelial-to-mesenchymal transition phenotype via targeting ADAM17 in oral squamous cell carcinoma. J Cell Mol Med 23: 4913–4920. 31207072 10.1111/jcmm.14107 PMC6653679

[92] Zang C. S., Huang H. T., Qiu J., Sun J., Ge R. F., and Jiang L. W.. 2020. MiR-224–5p targets EGR2 to promote the development of papillary thyroid carcinoma. Eur Rev Med Pharmacol Sci 24: 4890–4900. 32432752 10.26355/eurrev_202005_21178

[93] Hsiao S. Y., Weng S. M., Hsiao J. R., Wu Y. Y., Wu J. E., Tung C. H., Shen W. L., Sun S. F., Huang W. T., Lin C. Y., Chen S. H., Hong T. M., Chen Y. L., and Chang J. Y.. 2023. MiR-455–5p suppresses PDZK1IP1 to promote the motility of oral squamous cell carcinoma and accelerate clinical cancer invasion by regulating partial epithelial-to-mesenchymal transition. J Exp Clin Cancer Res 42: 40. 36737832 10.1186/s13046-023-02597-1 PMC9896797

[94] Meng C., Liu K., Cai X., and Chen Y.. 2021. Mechanism of miR-455–3 in suppressing epithelial-mesenchymal transition and angiogenesis of non-small cell lung cancer cells. Cell Stress Chaperones 27: 107–117. 35064898 10.1007/s12192-022-01254-4 PMC8943084

[95] Chen Y., Sun D., Shang D., Jiang Z., Miao P., and Gao J.. 2022. miR-223–3p alleviates TGF-beta-induced epithelial-mesenchymal transition and extracellular matrix deposition by targeting SP3 in endometrial epithelial cells. Open Med (Wars) 17: 518–526. 35350836 10.1515/med-2022-0424 PMC8919841

[96] Ke Y., Zhao W., Xiong J., and Cao R.. 2013. miR-149 Inhibits Non-Small-Cell Lung Cancer Cells EMT by Targeting FOXM1. Biochem Res Int 2013: 506731. 23762558 10.1155/2013/506731 PMC3671264

[97] Wang J., and Liu L.. 2021. MiR-149–3p promotes the cisplatin resistance and EMT in ovarian cancer through downregulating TIMP2 and CDKN1A. J Ovarian Res 14: 165. 34798882 10.1186/s13048-021-00919-5 PMC8605569

[98] Sun Y., Yi Y., Gan S., Ye R., Huang C., Li M., Huang J., and Guo Y.. 2021. miR-574–5p mediates epithelial-mesenchymal transition in small cell lung cancer by targeting vimentin via a competitive endogenous RNA network. Oncol Lett 21: 459. 33907569 10.3892/ol.2021.12720 PMC8063265

[99] Zhang K. J., Hu Y., Luo N., Li X., Chen F. Y., Yuan J. Q., and Guo L.. 2020. miR-574–5p attenuates proliferation, migration and EMT in triple-negative breast cancer cells by targeting BCL11A and SOX2 to inhibit the SKIL/TAZ/CTGF axis. Int J Oncol 56: 1240–1251. 32319565 10.3892/ijo.2020.4995

[100] Wang C., Zhang W., Zhang L., Chen X., Liu F., Zhang J., Guan S., Sun Y., Chen P., Wang D., Un Nesa E., Cheng Y., and Yousef G. M.. 2016. miR-146a-5p mediates epithelial-mesenchymal transition of oesophageal squamous cell carcinoma via targeting Notch2. Br J Cancer 115: 1548–1554. 27832663 10.1038/bjc.2016.367 PMC5155362

[101] Wang F., Ye L. J., Wang F. J., Liu H. F., and Wang X. L.. 2020. miR-146a promotes proliferation, invasion, and epithelial-to-mesenchymal transition in oral squamous carcinoma cells. Environ Toxicol 35: 1050–1057. 32469461 10.1002/tox.22941

[102] Liang Y., Li S., and Tang L.. 2021. MicroRNA 320, an Anti-Oncogene Target miRNA for Cancer Therapy. Biomedicines 9. 10.3390/biomedicines9060591 PMC8224659 34071109

[103] Liu R. Y., Zeng Y., Lei Z., Wang L., Yang H., Liu Z., Zhao J., and Zhang H. T.. 2014. JAK/STAT3 signaling is required for TGF-beta-induced epithelial-mesenchymal transition in lung cancer cells. Int J Oncol 44: 1643–1651. 24573038 10.3892/ijo.2014.2310

[104] Gregory P. A., Bracken C. P., Smith E., Bert A. G., Wright J. A., Roslan S., Morris M., Wyatt L., Farshid G., Lim Y. Y., Lindeman G. J., Shannon M. F., Drew P. A., Khew-Goodall Y., and Goodall G. J.. 2011. An autocrine TGF-beta/ZEB/miR-200 signaling network regulates establishment and maintenance of epithelial-mesenchymal transition. Mol Biol Cell 22: 1686–1698. 21411626 10.1091/mbc.E11-02-0103 PMC3093321

[105] Bettelli E., Carrier Y., Gao W., Korn T., Strom T. B., Oukka M., Weiner H. L., and Kuchroo V. K.. 2006. Reciprocal developmental pathways for the generation of pathogenic effector TH17 and regulatory T cells. Nature 441: 235–238. 16648838 10.1038/nature04753

[106] Guan T., Dominguez C. X., Amezquita R. A., Laidlaw B. J., Cheng J., Henao-Mejia J., Williams A., Flavell R. A., Lu J., and Kaech S. M.. 2018. ZEB1, ZEB2, and the miR-200 family form a counterregulatory network to regulate CD8(+) T cell fates. J Exp Med 215: 1153–1168. 29449309 10.1084/jem.20171352 PMC5881466

[107] Wang X. Y., Chen X. Y., Li J., Zhang H. Y., Liu J., and Sun L. D.. 2017. MiR-200a expression in CD4+ T cells correlates with the expression of Th17/Treg cells and relevant cytokines in psoriasis vulgaris: A case control study. Biomed Pharmacother 93: 1158–1164. 28738533 10.1016/j.biopha.2017.06.055

[108] Naghavian R., Ghaedi K., Kiani-Esfahani A., Ganjalikhani-Hakemi M., Etemadifar M., and Nasr-Esfahani M. H.. 2015. miR-141 and miR-200a, Revelation of New Possible Players in Modulation of Th17/Treg Differentiation and Pathogenesis of Multiple Sclerosis. PLoS One 10: e0124555. 25938517 10.1371/journal.pone.0124555 PMC4418573

[109] Bahmani L., Baghi M., Peymani M., Javeri A., and Ghaedi K.. 2021. MiR-141–3p and miR-200a-3p are involved in Th17 cell differentiation by negatively regulating RARB expression. Hum Cell 34: 1375–1387. 34086186 10.1007/s13577-021-00558-4

[110] Yu X., Fan X., Zhang X., Wei P., Zhou H., Liu D., and Chen B.. 2023. miR-429 inhibits the formation of an immunosuppressive microenvironment to counteract hepatocellular carcinoma immune escape by targeting PD-L1. Funct Integr Genomics 23: 312. 37775648 10.1007/s10142-023-01231-9

[111] Jebbawi F., Fayyad-Kazan H., Merimi M., Lewalle P., Verougstraete J. C., Leo O., Romero P., Burny A., Badran B., Martiat P., and Rouas R.. 2014. A microRNA profile of human CD8(+) regulatory T cells and characterization of the effects of microRNAs on Treg cell-associated genes. J Transl Med 12: 218. 25090912 10.1186/s12967-014-0218-x PMC4440568

[112] Cruz L. O., Hashemifar S. S., Wu C. J., Cho S., Nguyen D. T., Lin L. L., Khan A. A., and Lu L. F.. 2017. Excessive expression of miR-27 impairs Treg-mediated immunological tolerance. J Clin Invest 127: 530–542. 28067667 10.1172/JCI88415 PMC5272185

[113] Zhou Y., Li Y., Lu J., Hong X., and Xu L.. 2019. MicroRNA-30a controls the instability of inducible CD4+ Tregs through SOCS1. Mol Med Rep 20: 4303–4314. 31545427 10.3892/mmr.2019.10666

[114] Li J. Q., Tian J. M., Fan X. R., Wang Z. Y., Ling J., Wu X. F., Yang F. Y., and Xia Y. L.. 2020. miR-106b-5p induces immune imbalance of Treg/Th17 in immune thrombocytopenic purpura through NR4A3/Foxp3 pathway. Cell Cycle 19: 1265–1274. 32323598 10.1080/15384101.2020.1746485 PMC7469554

[115] Jiao P., Wang X. P., Luoreng Z. M., Yang J., Jia L., Ma Y., and Wei D. W.. 2021. miR-223: An Effective Regulator of Immune Cell Differentiation and Inflammation. Int J Biol Sci 17: 2308–2322. 34239357 10.7150/ijbs.59876 PMC8241730

[116] Chang C. C., Zhang Q. Y., Liu Z., Clynes R. A., Suciu-Foca N., and Vlad G.. 2012. Downregulation of inflammatory microRNAs by Ig-like transcript 3 is essential for the differentiation of human CD8(+) T suppressor cells. J Immunol 188: 3042–3052. 22387553 10.4049/jimmunol.1102899

[117] Lu L. F., Boldin M. P., Chaudhry A., Lin L. L., Taganov K. D., Hanada T., Yoshimura A., Baltimore D., and Rudensky A. Y.. 2010. Function of miR-146a in controlling Treg cell-mediated regulation of Th1 responses. Cell 142: 914–929. 20850013 10.1016/j.cell.2010.08.012 PMC3049116

[118] Zhou Q., Haupt S., Kreuzer J. T., Hammitzsch A., Proft F., Neumann C., Leipe J., Witt M., Schulze-Koops H., and Skapenko A.. 2015. Decreased expression of miR-146a and miR-155 contributes to an abnormal Treg phenotype in patients with rheumatoid arthritis. Ann Rheum Dis 74: 1265–1274. 24562503 10.1136/annrheumdis-2013-204377

[119] Li C., Ebert P. J., and Li Q. J.. 2013. T cell receptor (TCR) and transforming growth factor beta (TGF-beta) signaling converge on DNA (cytosine-5)-methyltransferase to control forkhead box protein 3 (foxp3) locus methylation and inducible regulatory T cell differentiation. J Biol Chem 288: 19127–19139. 23687305 10.1074/jbc.M113.453357 PMC3696685

[120] Yang H. Y., Barbi J., Wu C. Y., Zheng Y., Vignali P. D., Wu X., Tao J. H., Park B. V., Bandara S., Novack L., Ni X., Yang X., Chang K. Y., Wu R. C., Zhang J., Yang C. W., Pardoll D. M., Li H., and Pan F.. 2016. MicroRNA-17 Modulates Regulatory T Cell Function by Targeting Co-regulators of the Foxp3 Transcription Factor. Immunity 45: 83–93. 27438767 10.1016/j.immuni.2016.06.022 PMC4957244

[121] de Kouchkovsky D., Esensten J. H., Rosenthal W. L., Morar M. M., Bluestone J. A., and Jeker L. T.. 2013. microRNA-17–92 regulates IL-10 production by regulatory T cells and control of experimental autoimmune encephalomyelitis. J Immunol 191: 1594–1605. 23858035 10.4049/jimmunol.1203567 PMC4160833

[122] Skinner J. P., Keown A. A., and Chong M. M.. 2014. The miR-17 approximately 92a cluster of microRNAs is required for the fitness of Foxp3+ regulatory T cells. PLoS One 9: e88997. 24523948 10.1371/journal.pone.0088997 PMC3921252

[123] Dong L., Wang X., Tan J., Li H., Qian W., Chen J., Chen Q., Wang J., Xu W., Tao C., and Wang S.. 2014. Decreased expression of microRNA-21 correlates with the imbalance of Th17 and Treg cells in patients with rheumatoid arthritis. J Cell Mol Med 18: 2213–2224. 25164131 10.1111/jcmm.12353 PMC4224555

[124] Li D., Du X., Zhu M., Yang S., and Zhao W.. 2020. MiR-374b-5p Regulates T Cell Differentiation and Is Associated with rEg.P29 Immunity. Biomed Res Int 2020: 8024763. 32908913 10.1155/2020/8024763 PMC7463394

[125] Sun R., Zhang P. P., Weng X. Q., Gao X. D., Huang C. X., Wang L., Hu X. X., Xu P. P., Cheng L., Jiang L., Fu D., Qu B., Zhao Y., Feng Y., Dou H. J., Zheng Z., and Zhao W. L.. 2022. Therapeutic targeting miR130b counteracts diffuse large B-cell lymphoma progression via OX40/OX40L-mediated interaction with Th17 cells. Signal Transduct Target Ther 7: 80. 35301282 10.1038/s41392-022-00895-2 PMC8931122

[126] Liu S. Q., Jiang S., Li C., Zhang B., and Li Q. J.. 2014. miR-17–92 cluster targets phosphatase and tensin homology and Ikaros Family Zinc Finger 4 to promote TH17-mediated inflammation. J Biol Chem 289: 12446–12456. 24644282 10.1074/jbc.M114.550723 PMC4007439

[127] Honardoost M. A., Naghavian R., Ahmadinejad F., Hosseini A., and Ghaedi K.. 2015. Integrative computational mRNA-miRNA interaction analyses of the autoimmune-deregulated miRNAs and well-known Th17 differentiation regulators: An attempt to discover new potential miRNAs involved in Th17 differentiation. Gene 572: 153–162. 26307197 10.1016/j.gene.2015.08.043

[128] Wei Y., Chen S., Sun D., Li X., Wei R., Li X., and Nian H.. 2019. miR-223–3p promotes autoreactive T(h)17 cell responses in experimental autoimmune uveitis (EAU) by inhibiting transcription factor FOXO3 expression. FASEB J 33: 13951–13965. 31645142 10.1096/fj.201901446R

[129] Angelou C. C., Wells A. C., Vijayaraghavan J., Dougan C. E., Lawlor R., Iverson E., Lazarevic V., Kimura M. Y., Peyton S. R., Minter L. M., Osborne B. A., Pobezinskaya E. L., and Pobezinsky L. A.. 2019. Differentiation of Pathogenic Th17 Cells Is Negatively Regulated by Let-7 MicroRNAs in a Mouse Model of Multiple Sclerosis. Front Immunol 10: 3125. 32010153 10.3389/fimmu.2019.03125 PMC6978752

[130] Qu X., Zhou J., Wang T., Han J., Ma L., Yu H., Geng D., Fan H., Zhang Q., Hua F., and Yao R.. 2016. MiR-30a inhibits Th17 differentiation and demyelination of EAE mice by targeting the IL-21R. Brain Behav Immun 57: 193–199. 27006279 10.1016/j.bbi.2016.03.016

[131] Zhu E., Wang X., Zheng B., Wang Q., Hao J., Chen S., Zhao Q., Zhao L., Wu Z., and Yin Z.. 2014. miR-20b suppresses Th17 differentiation and the pathogenesis of experimental autoimmune encephalomyelitis by targeting RORgammat and STAT3. J Immunol 192: 5599–5609. 24842756 10.4049/jimmunol.1303488

[132] Chen J., Shi X., Deng Y., Dang J., Liu Y., Zhao J., Liang R., Zeng D., Wu W., Xiong Y., Yuan J., Chen Y., Wang J., Lin W., Chen X., Huang W., Olsen N., Pan Y., Fu Q., and Zheng S. G.. 2024. miRNA-148a-containing GMSC-derived EVs modulate Treg/Th17 balance via IKKB/NF-kappaB pathway and treat a rheumatoid arthritis model. JCI Insight 9. 10.1172/jci.insight.177841 PMC11141912 38652539

[133] Pacholewska A., Kraft M. F., Gerber V., and Jagannathan V.. 2017. Differential Expression of Serum MicroRNAs Supports CD4(+) T Cell Differentiation into Th2/Th17 Cells in Severe Equine Asthma. Genes (Basel) 8. 10.3390/genes8120383 PMC5748701 29231896

[134] Cao Y., Wang Z., Yan Y., Ji L., He J., Xuan B., Shen C., Ma Y., Jiang S., Ma D., Tong T., Zhang X., Gao Z., Zhu X., Fang J. Y., Chen H., and Hong J.. 2021. Enterotoxigenic Bacteroidesfragilis Promotes Intestinal Inflammation and Malignancy by Inhibiting Exosome-Packaged miR-149–3p. Gastroenterology 161: 1552–1566 e1512. 34371001 10.1053/j.gastro.2021.08.003

[135] Ahmadian-Elmi M., Bidmeshki Pour A., Naghavian R., Ghaedi K., Tanhaei S., Izadi T., and Nasr-Esfahani M. H.. 2016. miR-27a and miR-214 exert opposite regulatory roles in Th17 differentiation via mediating different signaling pathways in peripheral blood CD4+ T lymphocytes of patients with relapsing-remitting multiple sclerosis. Immunogenetics 68: 43–54. 26563334 10.1007/s00251-015-0881-y

[136] Cho S., Wu C. J., Yasuda T., Cruz L. O., Khan A. A., Lin L. L., Nguyen D. T., Miller M., Lee H. M., Kuo M. L., Broide D. H., Rajewsky K., Rudensky A. Y., and Lu L. F.. 2016. miR-23 approximately 27 approximately 24 clusters control effector T cell differentiation and function. J Exp Med 213: 235–249. 26834155 10.1084/jem.20150990 PMC4749926

[137] Akimniyazova A., Pyrkova A., Uversky V., and Ivashchenko A.. 2021. Predicting Associations of miRNAs and Candidate Gastric Cancer Genes for Nanomedicine. Nanomaterials (Basel) 11. 10.3390/nano11030691 PMC8000878 33801990

[138] Hironaka-Mitsuhashi A., Otsuka K., Gailhouste L., Sanchez Calle A., Kumazaki M., Yamamoto Y., Fujiwara Y., and Ochiya T.. 2020. MiR-1285–5p/TMEM194A axis affects cell proliferation in breast cancer. Cancer Sci 111: 395–405. 31854049 10.1111/cas.14287 PMC7004531

[139] Zhang J., Li X., Yang J., and Zhang Y.. 2022. MiR-1254 suppresses the proliferation and invasion of cervical cancer cells by modulating CD36. J Transl Med 19: 531. 36008842 10.1186/s12967-022-03582-6 PMC9413884

[140] Zhao M. C., Zhang M. M., Li T., Tao Z. H., Du Y. Q., Wang L. P., Zhang J., Wang B. Y., and Hu X. C.. 2020. MiR-566 protects the malignant progression of breast cancer by negatively regulating WNT6. Eur Rev Med Pharmacol Sci 24: 6185–6194. 32572884 10.26355/eurrev_202006_21514

[141] Zhou J., Zhang M., Huang Y., Feng L., Chen H., Hu Y., Chen H., Zhang K., Zheng L., and Zheng S.. 2015. MicroRNA-320b promotes colorectal cancer proliferation and invasion by competing with its homologous microRNA-320a. Cancer Lett 356: 669–675. 25458952 10.1016/j.canlet.2014.10.014 PMC4397650

[142] Pastor-Fernandez G., Mariblanca I. R., and Navarro M. N.. 2020. Decoding IL-23 Signaling Cascade for New Therapeutic Opportunities. Cells 9. 10.3390/cells9092044 PMC7563346 32906785

[143] Yeruva L., Pouncey D. L., Eledge M. R., Bhattacharya S., Luo C., Weatherford E. W., Ojcius D. M., and Rank R. G.. 2017. MicroRNAs Modulate Pathogenesis Resulting from Chlamydial Infection in Mice. Infect Immun 85. 10.1128/IAI.00768-16 PMC5203655 27799333

[144] Yeruva L., Bowlin A. K., Spencer N., Maurelli A. T., and Rank R. G.. 2015. Chlamydial variants differ in ability to ascend the genital tract in the guinea pig model of chlamydial genital infection. Infect Immun 83: 3176–3183. 26015484 10.1128/IAI.00532-15 PMC4496626

[145] Rajeeve K., Das S., Prusty B. K., and Rudel T.. 2018. Chlamydia trachomatis paralyses neutrophils to evade the host innate immune response. Nat Microbiol 3: 824–835. 29946164 10.1038/s41564-018-0182-y

